# Accumulation and regulation of anthocyanins in white and purple Tibetan Hulless Barley (*Hordeum vulgare* L. var. *nudum* Hook. f.) revealed by combined de novo transcriptomics and metabolomics

**DOI:** 10.1186/s12870-022-03699-2

**Published:** 2022-08-04

**Authors:** Xiaohua Yao, Youhua Yao, Likun An, Xin Li, Yixiong Bai, Yongmei Cui, Kunlun Wu

**Affiliations:** 1 Qinghai University, Xining, 810016 China; 2 Qinghai Academy of Agricultural and Forestry Sciences, Xining, 810016 China; 3 Qinghai Key Laboratory of Hulless Barley Genetics and Breeding, Xining, 810016 China; 4 Qinghai Subcenter of National Hulless Barley Improvement, Xining, 810016 China; 5 Laboratory for Research and Utilization of Qinghai Tibet Plateau Germplasm Resources, Xining, 810016 China

**Keywords:** Tibetan Hulless Barley, Seedcoat color, Transcriptomic and metabolomic, Proanthocyanin-anthocyanin biosynthesis, *ANS*

## Abstract

**Background:**

Colored barley, which may have associated human health benefits, is more desirable than the standard white variety, but the metabolites and molecular mechanisms underlying seedcoat coloration remain unclear.

**Results:**

Here, the development of Tibetan hulless barley was monitored, and 18 biological samples at 3 seedcoat color developmental stages were analyzed by transcriptomic and metabolic assays in Nierumuzha (purple) and Kunlun10 (white). A total of 41 anthocyanin compounds and 4186 DEGs were identified. Then we constructed the proanthocyanin-anthocyanin biosynthesis pathway of Tibetan hulless barley, including 19 genes encoding structural enzymes in 12 classes (*PAL*, *C4H*, *4CL*, *CHS*, *CHI*, *F3H*, *F3’H*, *DFR*, *ANS*, *ANR*, *GT*, and *ACT*). 11 DEGs other than *ANR* were significantly upregulated in Nierumuzha as compared to Kunlun10, leading to high levels of 15 anthocyanin compounds in this variety (more than 25 times greater than the contents in Kunlun10). *ANR* was significantly upregulated in Kunlun10 as compared to Nierumuzha, resulting in higher contents of three anthocyanins compounds (more than 5 times greater than the contents in Nierumuzha). In addition, 22 TFs, including *MYBs*, *bHLHs*, *NACs*, *bZips*, and *WD40s*, were significantly positively or negatively correlated with the expression patterns of the structural genes. Moreover, comparisons of homologous gene sequences between the two varieties identified 61 putative SNPs in 13 of 19 structural genes. A nonsense mutation was identified in the coding sequence of the *ANS* gene in Kunlun10. This mutation might encode a nonfunctional protein, further reducing anthocyanin accumulation in Kunlun10. Then we identified 3 modules were highly specific to the Nierumuzha (purple) using WGCNA. Moreover, 12 DEGs appeared both in the putative proanthocyanin-anthocyanin biosynthesis pathway and the protein co-expression network were obtained and verified.

**Conclusion:**

Our study constructed the proanthocyanin-anthocyanin biosynthesis pathway of Tibetan hulless barley. A series of compounds, structural genes and TFs responsible for the differences between purple and white hulless barley were obtained in this pathway. Our study improves the understanding of the molecular mechanisms of anthocyanin accumulation and biosynthesis in barley seeds. It provides new targets for the genetic improvement of anthocyanin content and a framework for improving the nutritional quality of barley.

**Supplementary Information:**

The online version contains supplementary material available at 10.1186/s12870-022-03699-2.

## Background

Tibetan hulless barley (*Hordeum vulgare* L. var. *nudum* Hook. f.), family Gramineae, is a barley variety that is highly genetically similar to barley (*Hordeum vulgare* L.) [1]. Because the lemma and palea of hulless barley grains are separated from the caryopsis at maturity, it is also known as naked barley or “qingke” on the Tibetan plateau, China [2]. Qingke is the most distinctive crop on the Tibetan plateau and is mainly found in the alpine zone at altitudes above 3000 m [3]. Tibetan hulless barley is used extensively in alcohol brewing, feeds, and food processing [4].

The seedcoat of the barley grain may be a variety of colors including white, blue, purple, and black [5]. Colored barley varieties have been shown to have more health benefits than the standard (white) variety because the colored varieties produce pearling fractions that are rich in bioactive compounds, such as anthocyanins [6]. Anthocyanins are the main chromophores that cause red, blue, and purple coloration in plant tissues such as flowers, fruits, and grains [7, 8]. Mullick et al. surveyed the anthocyanin content of eight barley varieties with grains of four different colors (white, blue, purple, and black) and found that anthocyanins were present in grains of all colors except white [9]. Zeng et al. reported a comprehensive metabolite profiling using 196 diverse qingke and barley accessions and obtained some flavonols and anthocyanins in the UV-B adaptation of qingke [10]. However, they did not identify the differences in anthocyanins involved in the synthesis of white and purple Tibetan hulless barley seedcoats.

Six anthocyanin compounds are commonly found in plants: pelargonidin, cyanidin, delphinidin, peonidin, petunidin, and malvidin; peonidin is formed by the methylation of cyanidin, while petunidin and malvidin are derived from delphinidin via varying degrees of methylation [7, 11]. Anthocyanins are physicochemically unstable and are only modified by glycosylation on the C3 or C5 backbone, resulting in the formation of stable anthocyanosides that are stored in the vesicles and thus color the plant part [12, 13].

The anthocyanin metabolism is a complex process that involves a variety of enzymes, including chalcone synthase (CHS), chalcone isomerase (CHI), dihydroflavonol-3-hydrogenase (F3H), dihydroflavonol-3’-hydrogenase (F3’H), dihydroflavonol-3’-5’-hydrogenase (F3′5’H), dioxanonol-4-reductase (DFR), and anthocyanidin synthase (ANS) [14]. Many of the genes encoding these enzymes were characterized in *Arabidopsis thaliana* using various mutants [15]. Alterations in the function or expression pattern of these structural genes can directly affect anthocyanin synthesis; for example, some *CHS* gene mutations prevent anthocyanin synthesis, resulting in white petal phenotypes in petunias [16] and violets [17], while transfer of the while transfer of the lychee acyltransferase gene *LcGST4* into mutant tt19, an *Arabidopsis* glutathione S-transferase GST mutant, restored anthocyanin synthesis in the hypocotyl [18]. Transformation of the antisense gene of *CHS* into petunia similarly changed the color from purplish red to pink or even white [19]. The transcription factors basic helix-loop-helix (bHLH), v-myb avian myeloblastosis viral oncogene homolog (*MYB*), and *WD40* are important regulators of the expression of structural genes in the anthocyanin metabolism pathway [20] in species such as *Petunia hybrid* [21]. Overexpression of *MYB1* in *Petunia hybrida* upregulated the anthocyanin synthesis-associated genes *ANS* and *DFR,* increasing anthocyanin content [22]. The regulation of anthocyanin synthesis by *bHLH* transcription factors has been demonstrated in a number of plants, including *Arabidopsis*, goldenseal, chrysanthemum, and begonia [23, 24].

In barley, *CHS* was upregulated in response to UV or pathogen stress [25], and the genes encoding F3’H and F3′5’H exhibited tissue-specific expression patterns [26]. In barley mutants unable to synthesize anthocyanin and proanthocyanidin, the expression of *CHS, DFR,* and *F3’H* was suppressed [27]. Finally, nearly full-length cDNA clones of barley *Phenylalanine ammonia-lyase* (*PAL*) were shown to be highly similar to wheat and rice *PAL* sequences, and these barleys *PAL* genes were upregulated in response to stressors such as mercuric chloride and pathogenic fungi [28]. Gordeeva et al. revealed that the dominant alleles of both the *Ant1* and *Ant2* genes were required for anthocyanin accumulation in pericarp of barley [29]. Overexpression of *Ant1* led to anthocyanin accumulation in the pericarp and aleurone layer of transgenic barley grains [30]. However, it remains unclear which structural genes and transcription factors play the major role in seedcoat color formation of purple Tibetan hulless barley.

Recent high-throughput sequencing analyses, especially in combination with metabolome and transcriptome analysis, have characterized anthocyanin synthesis pathways in a variety of non-model plants such as asparagus, turnip, and grape [31–33]. The complete anthocyanin synthesis pathway of purple Tibetan hulless barley has not been reported. Here, we investigated differences in anthocyanin biosynthesis between the white and purple Tibetan hulless barley. The components of the anthocyanin biosynthetic pathways in the different cultivars were identified using Multiple Reaction Monitoring (MRM). Genes associated with anthocyanin biosynthesis and its regulations were identified using transcriptomics. Of these, the structural genes and transcription factors were further examined to construct a preliminary anthocyanin synthesis pathway specific to purple barley varieties. The results improve our understanding of the molecular mechanisms underlying anthocyanin biosynthesis in barley and provide valuable information for the genetic improvement of purple barley and other purple crops.

## Results

### Anthocyanin content in purple and white Tibetan hulless barley grains

**Fig. 1:**
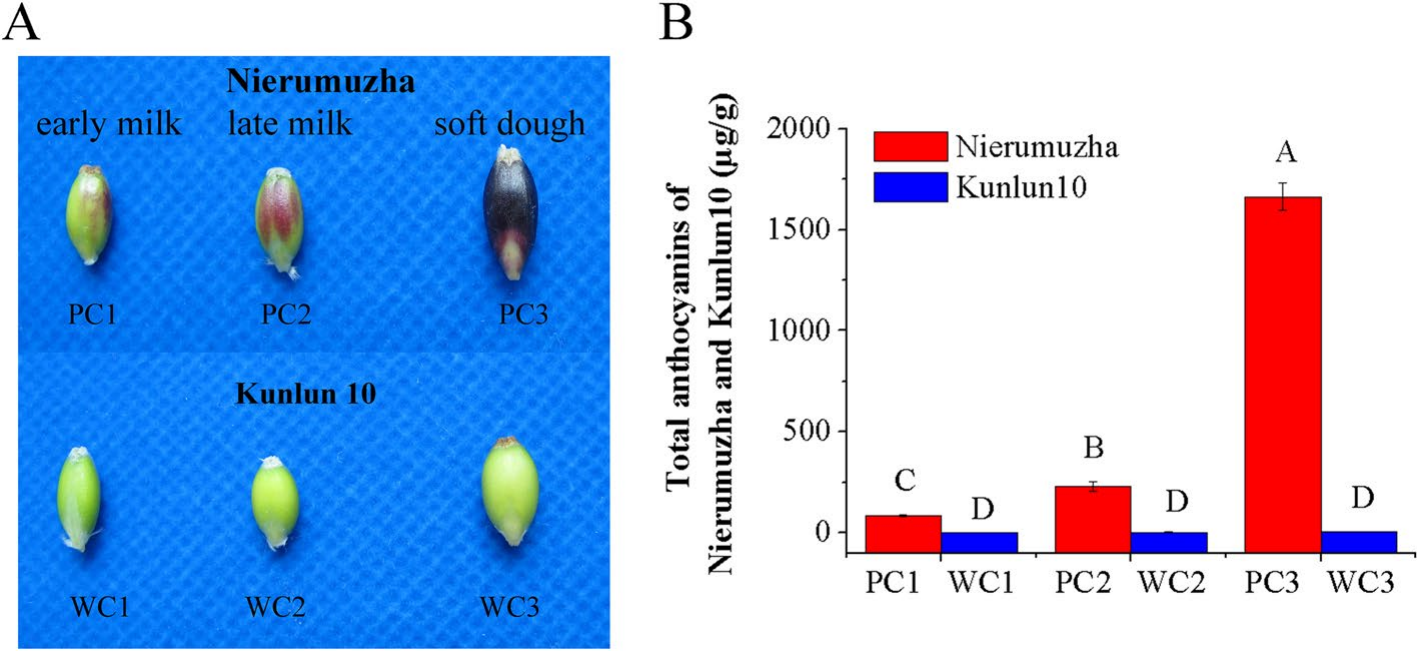
Changes in anthocyanin content of white (Kunlun 10) and purple (Nierumuzha) Tibetan hulless barley grains. Grains were analyzed at the early milk (PC1/WC1), late milk (PC2/WC2), and soft dough stages (PC3/WC3). **A** Grain phenotype. **B** Mean total anthocyanin content. Data are shown as the mean ± standard error (SE) of three biological replicates

We first compared total anthocyanin content between the seedcoats of the white (Kunlun10, control) and purple (Nierumuzha) Tibetan hulless barley grains (Fig. 1). Grain samples were collected at three different developmental stages: early milk, late milk, and soft dough (Fig. 1A). The total anthocyanin content of Kunlun10 remained low throughout the testing period (0.13–4.00 μg/g), and there were no significant differences in total anthocyanin content among the three stages in this variety (Fig. 1B). In contrast, total anthocyanin content in Nierumuzha was significantly higher than that in Kunlun10 initially (83.83 µg/g; *P* < 0.01), and increased significantly at each stage (*P* < 0.01) to peak at 1663.44 μg/g (Fig. 1B). Total anthocyanin content in Nierumuzha at the soft dough stage was about 20 times greater than that at the early milk stage and about 7 times greater than that at the late milk stage (Fig. 1B). Thus, there were substantial variety-specific differences in total anthocyanin content.

### Anthocyanin compounds in the seedcoats of the two Tibetan hulless varieties

**Fig. 2:**
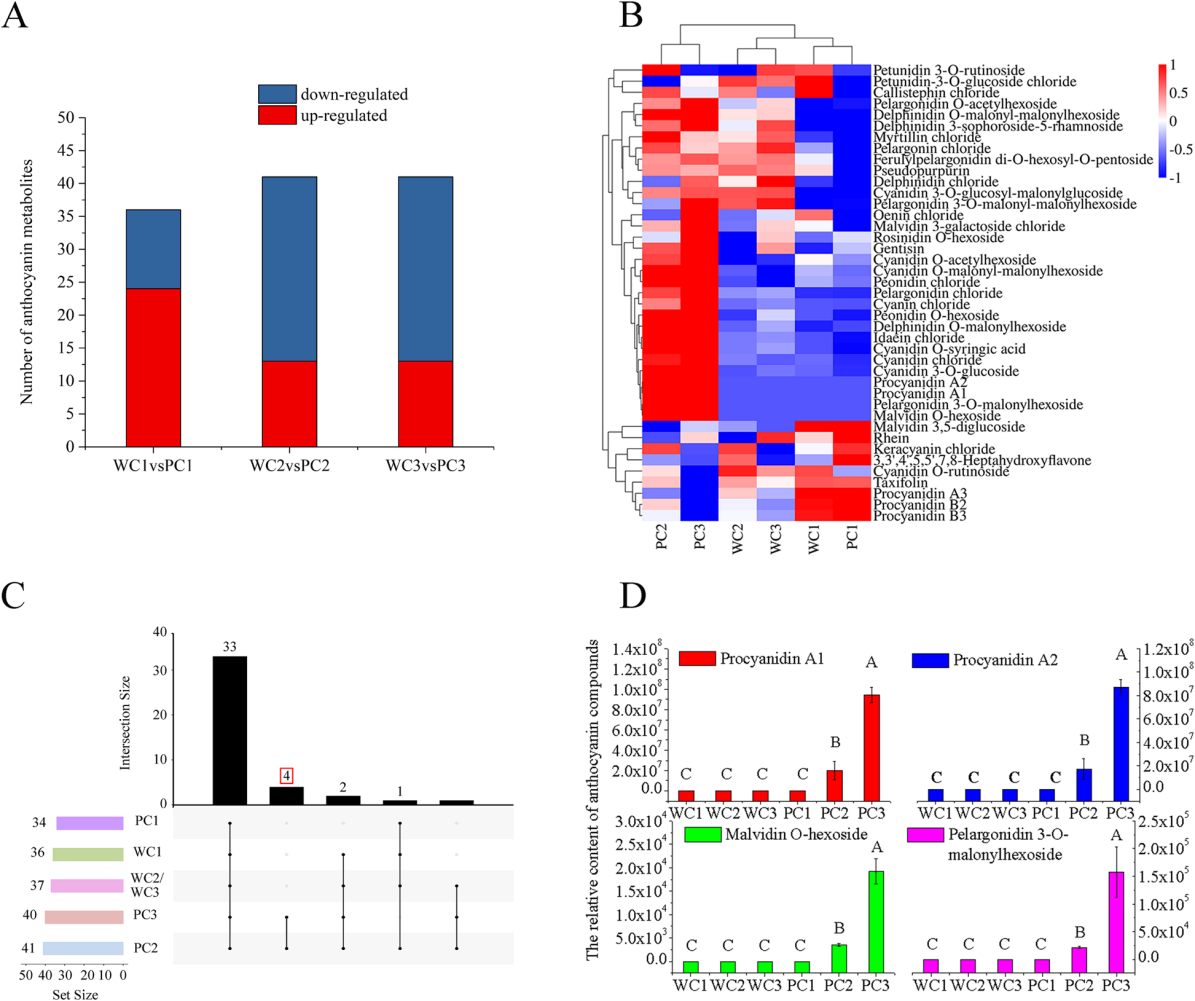
Differentially abundant anthocyanin compounds between Kunlun10 and purple Nierumuzha at three stages. **A** Numbers of differentially abundant anthocyanin compounds between varieties at each stage. **B** Heatmap showing the patterns of differential abundance across the 41 anthocyanin compounds at each stage. Cell colors correspond to the log10 magnitude of the difference in abundance (fold change values + 1): redder cells indicate higher abundance, and bluer cells indicate lower abundance. **C** Numbers of differentially accumulated anthocyanins shared and unique between the two varieties at the three developmental stages. **D** Content of four anthocyanin compounds absent from all stages of Kunlun10 and the early milk stage of Nierumuzha

Using MRM, we detected 41 anthocyanin compounds in the seedcoats of the two Tibetan hulless barley cultivars (Table S1; Fig. 2). The numbers of unique anthocyanin compounds were similar among each variety at each stage: early milk, late milk, and soft dough, 36, 37, and 37 compounds were detected in the seedcoat of Kunlun10, respectively, while 34, 41, and 40 compounds were detected in the seedcoat of Nierumuzha, respectively. Then, we identified significant differences in the abundances of 39 out of the 41 anthocyanin compounds between varieties at least one developmental stage; only cyanidin 3-O-glucosyl-malonylglucoside and delphinidin chloride did not differ significantly in abundance between varieties at any stage (Table S1). Four additional compounds of 41 anthocyanin compounds, Pseudopurpurin, Rhein, Taxifolin, and Gentisin, were predicted to have similar structures and molecular formulas to flavonoids and anthocyanins. Meanwhile, we found that the number of anthocyanin compounds in late milk stage and soft dough stage increased by 5 compared with that in early milk stage (Fig. 2A). Moreover, the contents of most anthocyanin compounds in the late milk and soft dough stages of Nirumuzha were significantly higher than those in the early milk stage and the three stages of Kunlun 10 (Fig. 2B). In particular, four anthocyanin compounds (Procyanidin A1, Procyanidin A2, Malvidin O-hexoside, and Pelargonidin 3-O-malonylhexoside) were abundant in the late milk and soft dough stages of Nierumuzha, but absent in the three stages of Kunlun10 and in the early milk stage of Nierumuzha (Fig. 2C and D). Many additional anthocyanin compounds, including Cyanidin O-malonyl-malonylhexoside and Peonidin O-hexoside, were significantly more abundant in the Nierumuzha seedcoat as compared to the Kunlun10 seedcoat (Table S1). These differentially abundant anthocyanin compounds might play an important role in the formation of the purple seedcoat of Nierumuzha.

### Sequencing and assembly of the transcriptomes of the two Tibetan hulless barleys

To better understand the relationships between anthocyanin-compound abundance and grain coloration, we generated RNA-seq data for the seedcoats of Kunlun10 and Nierumuzha at the three different developmental stages. In total, we generated 112.62 Gb clean data, an average of 6.26 Gb per sample. The Q20 ratio of each sample was 97.16–98.38%, and the Q30 was 92.57–94.99%. GC content was relatively consistent, at around 54%, across all samples. Across the six samples (PC1, PC2, PC3, WC1, WC2 and WC3), 37.34, 37.39, 36.53, 37.35, 36.35, and 36.76 million reads, respectively, were mapped to the reference barley (*H. vulgare*) genome in total, while 33.34, 33.17, 32.44, 33.09, 31.44, and 31.92 million reads, respectively, were uniquely mapped (Table S2).

### Differentially expressed genes (DEGs) between the two Tibetan hulless barleys at each developmental stage

**Fig. 3:**
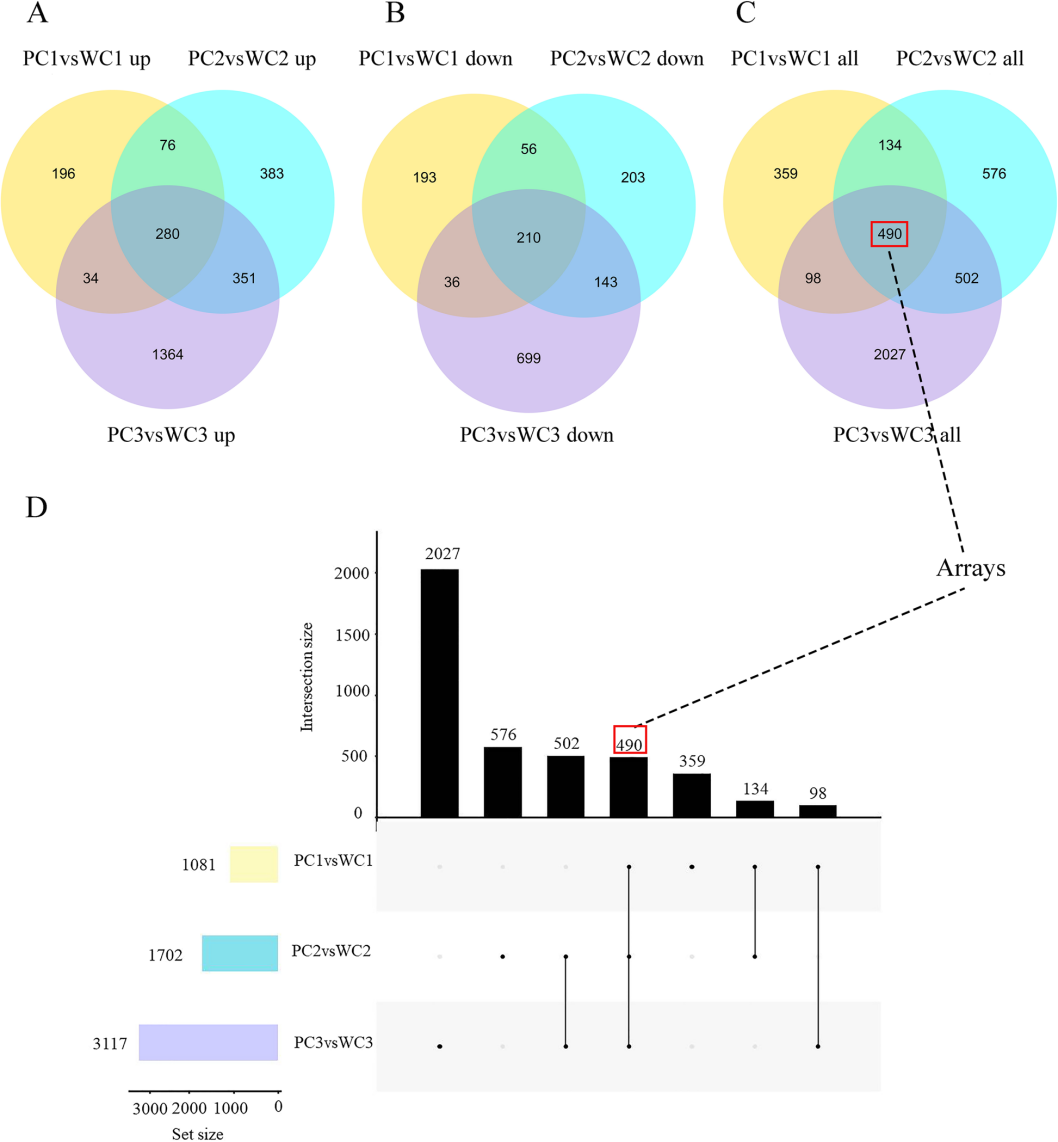
Identification of DEGs between Kunlun10 and Nierumuzha at three stages. **A–C** Venn diagrams showing DEGs shared and unique between the two barley varieties at each developmental stage: downregulated, upregulated, and all. **D** Veen analysis of DEGs among PC1vsWC1, PC2vsWC2 and PC2vsWC2

To identify DEGs between the purple- and white-grain Tibetan hulless barley, we compared fragments per kilobase per million (FPKM) values between the Kunlun10 and Nierumuzha transcriptomes at each developmental stage (Fig. 3). We considered genes significantly differentially expressed when |log2 (FoldChange)| was > 1 and the adjusted *P*-value was < 0.05. In Nierumuzha grains as compared to Kunlun10 grains, 1081 DEGs were identified at the early milk stage (586 upregulated and 495 downregulated), 1702 DEGs were identified at the late milk stage (1090 upregulated genes and 612 downregulated), and 3117 DEGs were identified at the soft dough stage (2029 upregulated and 1088 downregulated). The number of DEGs was greatest at the soft dough stage, suggesting that this stage might be most important for grain color formation. Across all three stages, 4186 DEGs were identified between Nierumuzha as compared to Kunlun10 (2684 upregulated and 1540 downregulated; Fig. 3A–C). Of these, 490 were differentially expressed between the two varieties across all three developmental stages (Fig. 3C–D).

Annotation of the DEGs against Swiss-Prot and various other databases identified 133 DEGs might be related to anthocyanin synthesis (of the total 4186; Table S3). Notably, we also identified one DEG (HORVU7Hr1G116630; Table S3) without functional annotation, which was significantly positively correlated with the formation of seedcoat color and was therefore included as a candidate gene for anthocyanin synthesis. After excluding DEGs with FPKM < 20 in all samples (PC1, PC2, PC3, WC1, WC2 and WC3), 40 DEGs related to anthocyanin synthesis were obtained. Of these, 35 DEGs were significantly upregulated in Nierumuzha at the late milk and soft dough stages as compared to Kunlun10 at all stages and to Nierumuzha at the early milk stage (Figure S1). The remaining five DEGs were significantly downregulated in Nierumuzha at the soft dough stage as compared to all other samples (Figure S1).

### DEGs encoding transcription factors (TFs)

**Fig. 4:**
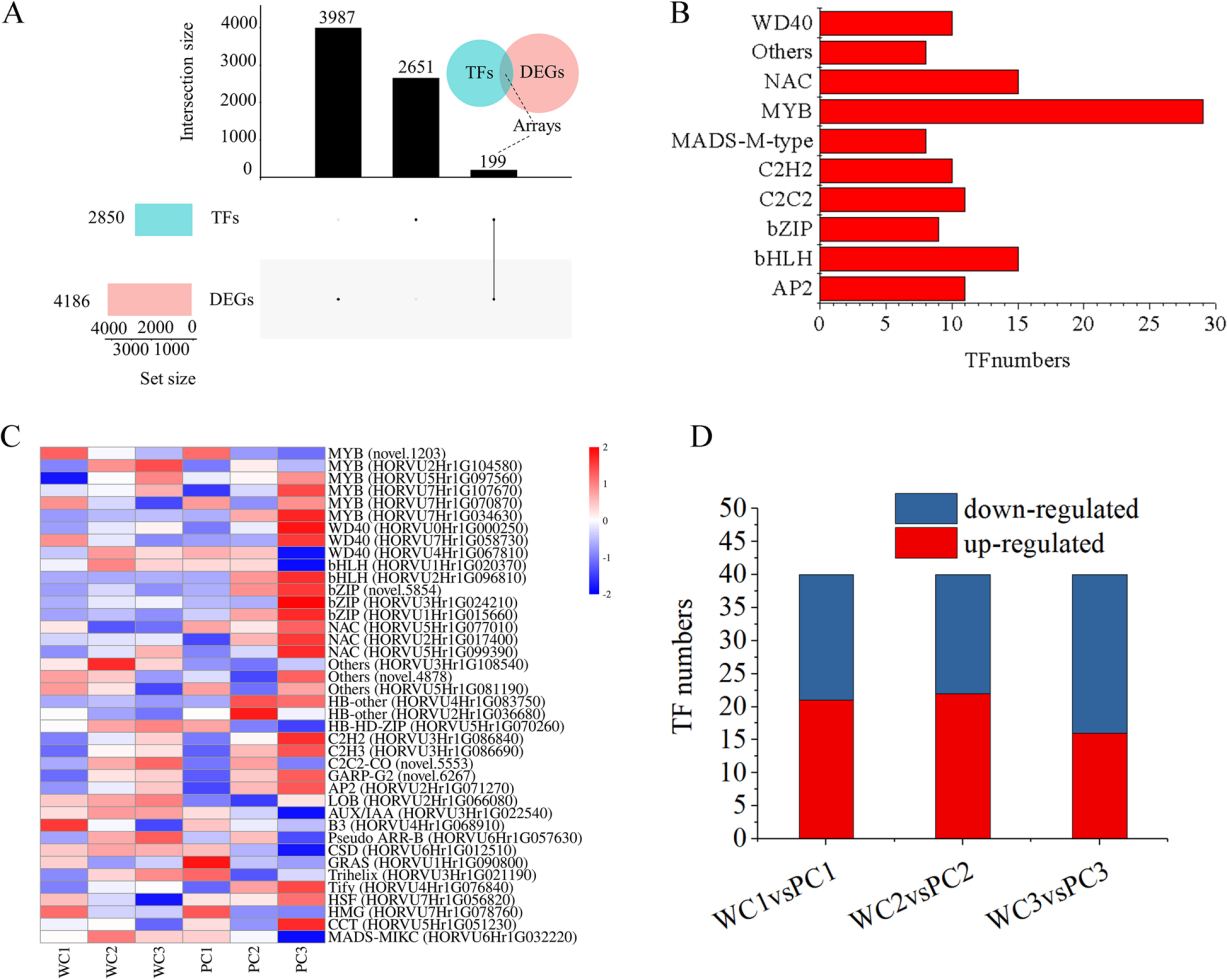
Genes differentially expressed between the two barley varieties encoding transcription factors (TFs). **A** Differentially expressed TFs. **B** Distribution of the identified differentially expressed TFs (Top 10). **C** Heatmap showing the 40 differentially expressed TFs between the two varieties at each stage. Cell colors correspond to the log10 magnitude of the difference in expression level [log10 (fold change values + 1)]: redder cells indicate upregulation, while bluer cells indicate downregulation. **D** Numbers of differentially expressed TFs between varieties at each stage

To identify TFs related to anthocyanin synthesis, we analyzed TFs with significant differences in expression between the Kunlun10 and Nierumuzha at eachdevelopmental stage (Fig. 4). Across the transcriptomes generated from each sample, 2850 genes encoding TFs were identified. Comparison with the DEGs showed that the genes encoding 199 of these TFs were differentially expressed (Fig. 4A). These 199 TFs primarily belonged to 44 TF families and may modulate global gene expression levels during the synthesis of anthocyanins (Fig. 4B). Across the 199 TFs, *MYB* TFs were the most common (29/199, 14.57%), followed by *bHLH* and *NAC* (both 15/199, 7.54%) (Fig. 4B). After excluding DEGs with FPKM < 20 in all samples (PC1, PC2, PC3, WC1, WC2 and WC3), 40 TFs associated with anthocyanin synthesis remained (Fig. 4C). Compared to Kunlun10 grains, 21 TFs were upregulated and 19 TFs were downregulated at the early milk stage, 22 TFs were upregulated and 18 TFs were downregulated at the late milk stage, and 16 TFs were upregulated and 24 TFs were downregulated at the soft dough stage in Nierumuzha (Fig. 4D). Therefore, these TFs might be important for the regulation of structural genes involved in the coloration of the Tibetan hulless barley seedcoat.

### Correlations between DEGs associated with anthocyanin synthesis and TFs or differentially abundant anthocyanin compounds

**Fig. 5:**
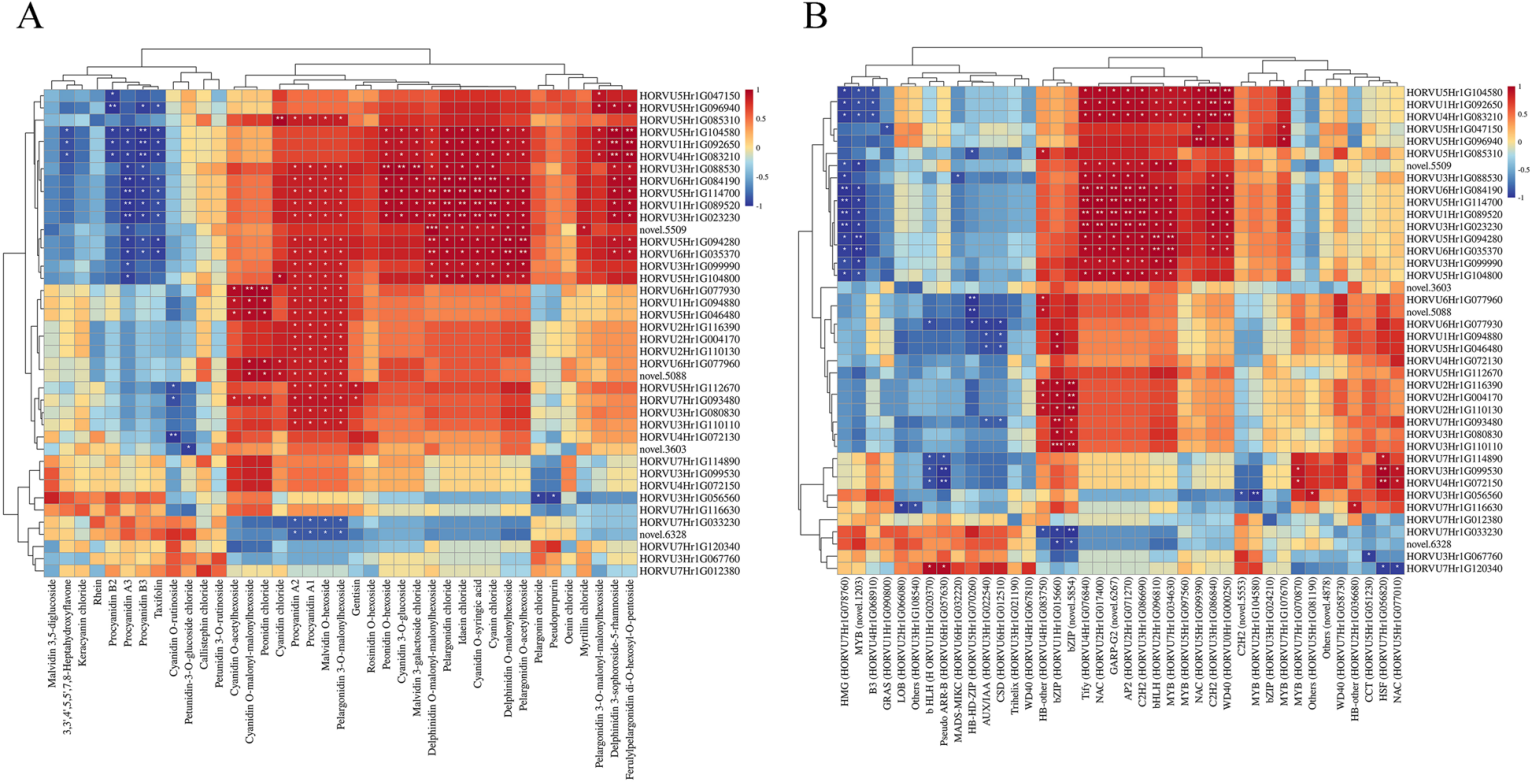
Heatmaps showing correlations between the DEGs and the anthocyanin compounds or TFs. **A** The DEGs associated with anthocyanin synthesis and the differentially abundant anthocyanin compounds. **B** The DEGs associated with anthocyanin synthesis and TFs. Redder cells correspond to stronger positive correlations, while bluer cells correspond to stronger negative correlations. Significant correlations in each cell are indicated using asterisks: *, *P* < 0.05; **, *P* < 0.01; ***, *P* < 0.001

We investigated the correlation between 40 DEGs associated with anthocyanin synthesis and 40 differential TFs or 39 differential anthocyanin compounds. Correlation analysis showed that 33 DEGs were significantly correlated with 32 anthocyanin compounds (Fig. 5A). In particular, the abundances of Procyanidin A1, Procyanidin A2, Malvidin O-hexoside, and Pelargonidin 3-O-malonylhexoside were significantly positively correlated with 22 DEGs (*P* < 0.05; *R* > 0.5) and significantly negatively correlated with 2 DEGs (*P* < 0.05; *R* <  − 0.5). Correlation analysis showed that 36 DEGs associated with anthocyanin synthesis were significantly correlated with 35 DEGs encoding TFs (Fig. 5B).

### A proposed model of the proanthocyanin-anthocyanin biosynthesis pathways of the white and purple hulless barleys

**Fig. 6:**
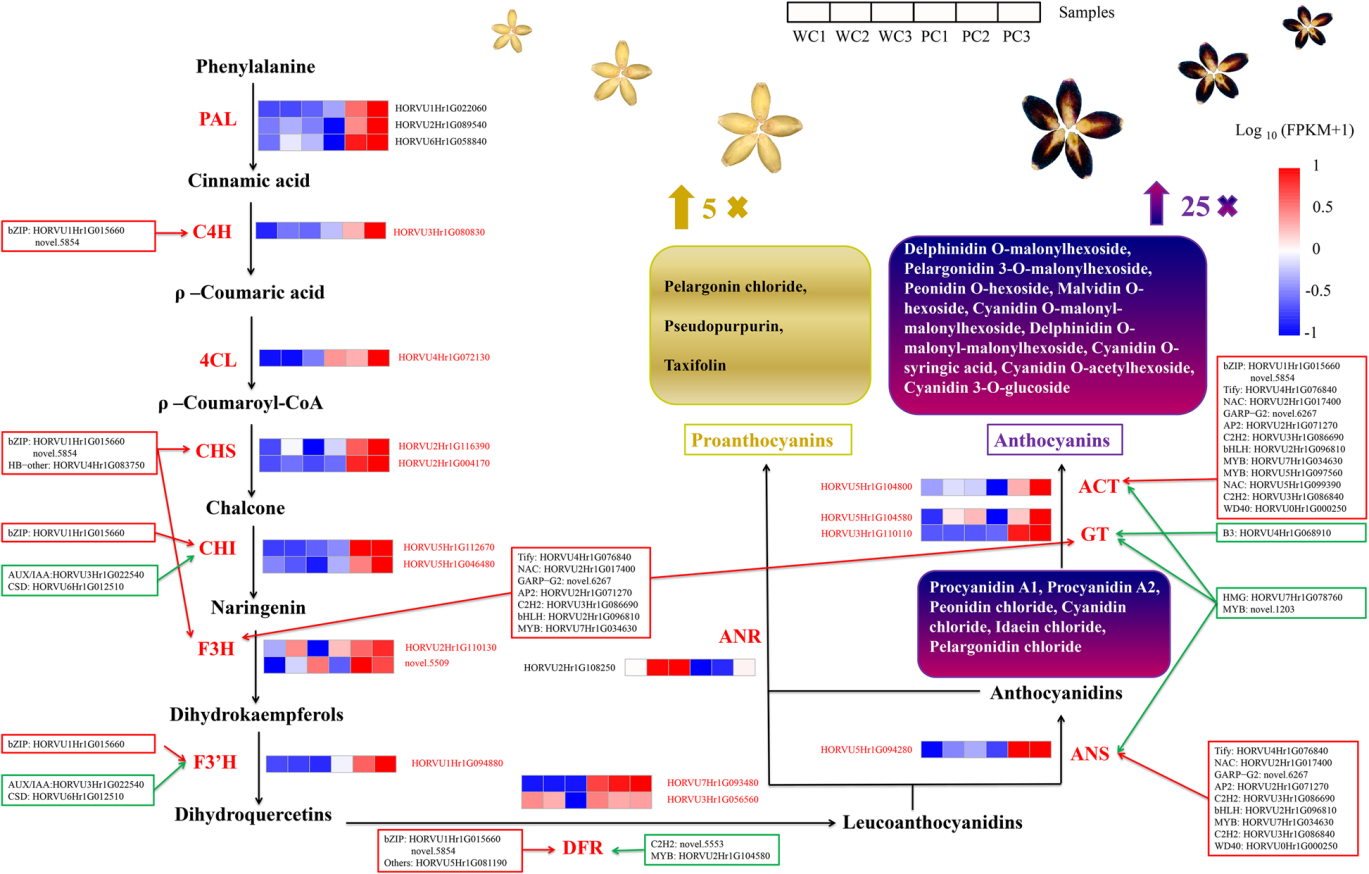
Proposed model of the molecular mechanisms and the proanthocyanin-anthocyanin biosynthesis pathways in Tibetan hulless barley. The structural enzymes are identified using large red capital letters; the encoding gene IDs are shown in small capital letters and are also in red if these genes are significantly differentially expressed between the two varieties. Red boxes and arrows correspond to TFs that are significantly positively correlated with, and thus may positively regulate, genes encoding structural enzymes. Green boxes and arrows correspond to TFs that are significantly negatively correlated with, and thus may negatively regulate, genes encoding structural enzymes

To predict the molecular mechanisms underlying the differences in seedcoat coloration between Kunlun10 and Nierumuzha, we first screened the 134 DEGs might be associated with anthocyanin biosynthesis to identify DEGs coding for enzymes involved in the proanthocyanin-anthocyanin biosynthesis pathways (Table S3). We successfully identified 19 DEGs encoding 12 classes of structural enzymes implicated in the proanthocyanin-anthocyanin biosynthesis pathways: *PAL* (HORVU1Hr1G022060, HORVU2Hr1G089540, and HORVU6Hr1G058840), *cinnamic acid 4-hydroxylase* (*C4H*; HORVU3Hr1G080830), *4-coumarate-CoA ligase* (*4CL*; HORVU4Hr1G072130), *CHS* (HORVU5Hr1G112670 and HORVU5Hr1G046480), *CHI* (HORVU2Hr1G116390 and HORVU2Hr1G004170), *F3H* (HORVU2Hr1G110130 and novel.5509), *F3’H* (HORVU1Hr1G094880), *DFR* (HORVU7Hr1G093480 and HORVU3Hr1G056560), *ANS* (HORVU5Hr1G094280), *anthocyanidin reductase* (*ANR*; HORVU2Hr1G108250), *UDP-glucosyltransferase* (*GT*; HORVU5Hr1G104580 and HORVU3Hr1G110110) and *acetyltransferase* (*ACT*; HORVU5Hr1G104800). Of these 19 DEGs, 11, encoding the structural enzymes PAL, C4H, 4CL, CHS, CHI, F3H, F3’H, DFR, ANS, GT, and ACT, were significantly upregulated in Nierumuzha as compared to Kunlun10, resulting in notably greater levels of 15 anthocyanins compounds in Nierumuzha (more than 25 times greater than the levels of these compounds in Kunlun10; Table S1). Conversely, *ANR* was significantly downregulated in Nierumuzha as compared to Kunlun10, resulting in lower contents of three anthocyanins compounds in Nierumuzha (levels in Kunlun10 were five times greater than those in Nierumuzha; Table S1). In conjunction with our anthocyanin-compound quantification, our results indicated that the six anthocyanins primarily responsible for the purple pigmentation of Nierumuzha were procyanidin A1, procyanidin A2, malvidin O-hexoside, pelargonidin 3-O-malonylhexoside, delphinidin O-malonyl-malonylhexoside, and peonidin chloride. In addition, using the results of correlation analysis between DEGs and TFs related to anthocyanin synthesis (Fig. 4B), we obtained 22 TFs that were significantly correlated with the expression of genes encoding these 19 structural proteins, including *MYB*, *bHLH*, *NAC*, *bZip*, and *WD40*. These TFs might thus participate in the regulation of the expression of these structural genes during grain color formation. Based on these results, we reconstructed the proanthocyanin-anthocyanin biosynthesis pathways leading to the different seedcoat coloration between the two Tibetan hulless barley varieties (Fig. 6).

### Identification of SNPs within the structural genes regulating differential seedcoat coloration in the white and purple hulless barleys

**Fig. 7:**
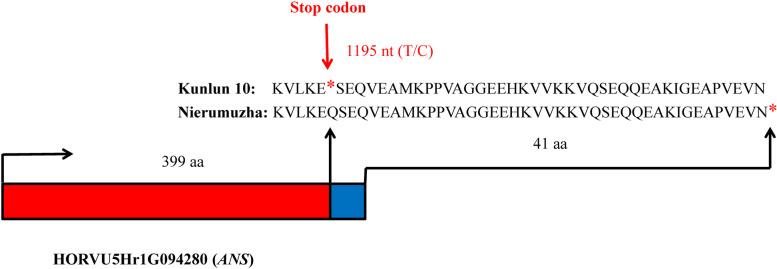
Point-nonsynonymous mutation at 1,195 of the coding sequences of ANS (HORVU5Hr1G094280) between Kunlun10 and Nierumuzha

We next identified SNPs between Kunlun10 and Nierumuzha in the 19 DEGs encoding the 12 structural enzymes that were implicated in the proanthocyanin-anthocyanin biosynthesis pathways. Sequence comparisons between the two Tibetan hulless barley varieties revealed 61 putative SNPs in 13 DEGs (Table S4). For example, the *ANS* gene (HORVU5Hr1G094280), which has a single coding sequence of 1,197 nucleotides, had a point-nonsynonymous mutation (T/C) at position 1,195 of the CDS (Fig. 7). This SNP had an allele depth (number of average reads) of 4/323 for Nierumuzha and 0/5 for Kunlun10. Then We verified the authenticity of this gene with PCR amplification. The result indicated that allele T, which corresponds to a stop codon in the resulting protein, was likely to be present only in Kunlun10, while allele C was apparently only present in Nierumuzha (Fig. 7).

### Identification of gene co-expression modules associated with barley seedcoat color using weighted correlation network analysis (WGCNA)

**Fig. 8:**
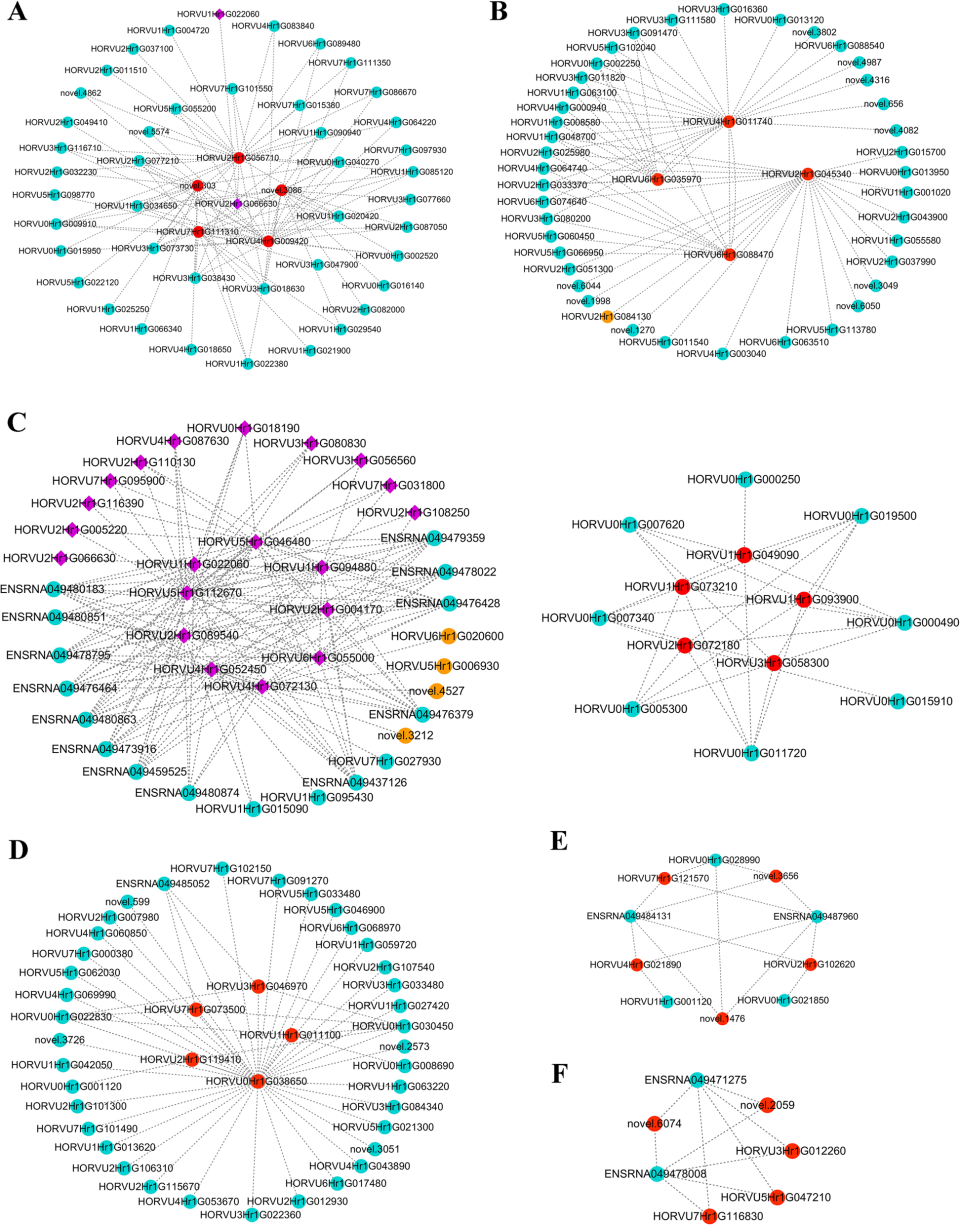
Gene co-expression networks and hub genes in the different modules. **A** Brown. **B** Yellow. **C** Turquoise. **D** Black. **E** Red. **F** Green. Red circles correspond to hub genes. Purple diamonds correspond to both genes associated with anthocyanin synthesis and genes from PPI network. Orange circles correspond to genes from PPI network

After removal of any genes that were unexpressed in either variety at any stage (FPKM = 0), the remaining 25,375 genes were used for WGCNA. A scale-free network was constructed to obtain 25 colored modules, corresponding to clusters of co-expressed genes (Figure S2). The cluster modules ranged in size from 49 genes (orange) to 5801 genes (turquoise). Across the 25 gene modules, three were highly specific to the Nierumuzha (purple) variety (|r|> 0.90, *P* < 0.05), each of which were significantly positively correlated with one stage of development: brown with the early milk stage (*r* = 0.96, *P* = 2e-3; Fig. 8A), yellow with the late milk stage (*r* = 0.92, *P* = 9e-3; Fig. 8B), and turquoise with the soft dough stage (*r* = 0.95, *P* = 4e-3; Fig. 8C). Similarly, three modules were highly specific to the Kunlun10 (white) variety (|r|> 0.90, *P* < 0.05) and were also significantly positively correlated with one stage of development: black with the early milk stage (*r* = 0.95, *P* = 4e-03; Fig. 8D), red with the late milk stage (*r* = 0.90, *P* = 0.02; Fig. 8E), and green with the soft dough stage (*r* = 0.95, *P* = 4e-03; Fig. 8F). A total of 10 genes associated with anthocyanin synthesis were found in six significantly correlated modules. The five genes with the highest linkage values across the six significantly correlated modules, as well as 10 anthocyanin synthesis-related genes, were selected as hub genes for the visualization of an interaction network (Fig. 8). A total of 22 genes related to anthocyanin synthesis were identified in the six modules, 20 of which were present in the turquoise module. We applied the interactions in the STRING protein interactions database for the analysis of protein–protein interactions (PPIs) encoded by DEGs (Table S5). The PPIs results showed that a total of 4 genes (orange circles) interacted with 22 genes (purple diamond) related to anthocyanin synthesis (Fig. 8A and C), and only one gene (HORVU2Hr1G045340) of the hub genes interacted with the other one (HORVU2Hr1G084130) (Fig. 8B).

### Validation of DEGs associated with proanthocyanin-anthocyanin biosynthesis

**Fig. 9:**
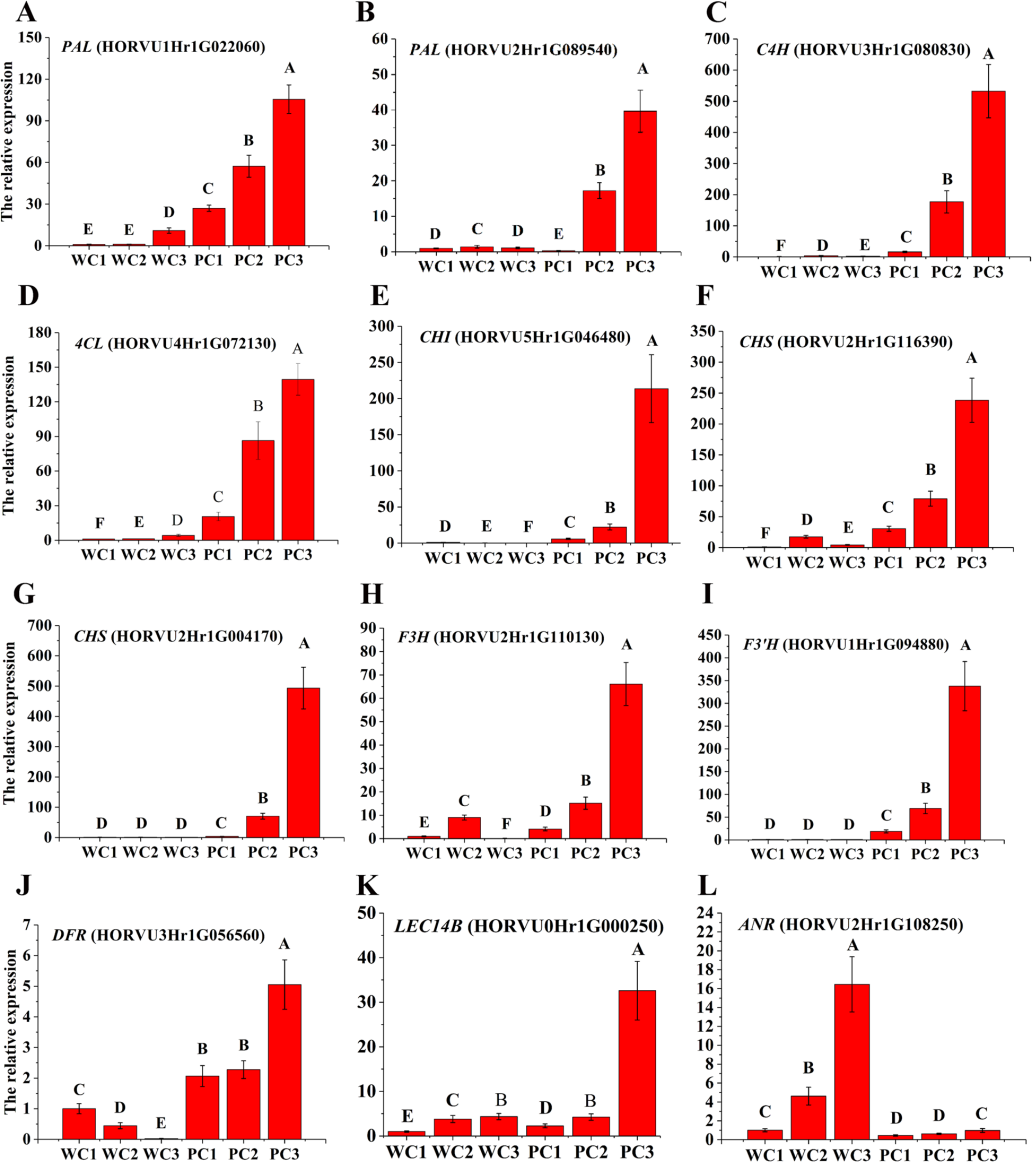
Expression patterns of the 11 structural genes and one transcription factor in Kunlun10 and Nierumuzha. The *TC139057* were used as internal gene. **A** The relative expression of *PAL* (HORVU1Hr1G022060). **B** The relative expression of *PAL* (HORVU2Hr1G089540). **C** The relative expression of *C4H* (HORVU3Hr1G080830). **D** The relative expression of *4CL* (HORVU4Hr1G072130). **E** The relative expression of *CHI* (HORVU5Hr1G046480). **F** The relative expression of *CHS* (HORVU2Hr1G116390). **G** The relative expression of and *CHS* (HORVU2Hr1G004170). **H** The relative expression of *F3H* (HORVU2Hr1G110130). **I** The relative expression of *F3’H* (HORVU1Hr1G094880). **J** The relative expression of *DFR* (HORVU3Hr1G056560). **K** The relative expression of *ANR* (HORVU2Hr1G108250). **L** The relative expression of WD40-like TF *LEC14B* (HORVU0Hr1G000250). The data displayed in the histograms are expressed as the means ± SD

We used quantitative real-time PCR (qRT-PCR) to validate the differential expression patterns of the RNA-Seq DEGs. We selected 11 DEGs encoding structural proteins including 2 *PAL* (HORVU1Hr1G022060 and HORVU2Hr1G089540), *C4H* (HORVU3Hr1G080830), *4CL* (HORVU4Hr1G072130) *CHI* (HORVU5Hr1G046480), 2 *CHS* (HORVU2Hr1G116390 and HORVU2Hr1G004170), *F3H* (HORVU2Hr1G110130), *F3’H* (HORVU1Hr1G094880), *DFR* (HORVU3Hr1G056560), *ANR* (HORVU2Hr1G108250) and one WD40-like TF *LEC14B* (HORVU0Hr1G000250) for validation; all 12 DEGs appeared both in the putative proanthocyanin-anthocyanin biosynthesis pathway (Fig. 6) and the protein co-expression network (Fig. 8). In the qRT-PCR analysis, 10 genes (2 *PALs*, *C4H*, *4CL*, *CHI*, 2 *CHSs*, *F3H*, *F3’H*, *DFR* and *LEC14B* were expressed at lower levels in Kunlun10 during all three stages of seedcoat color formation, whereas they were almost highly significantly up-regulated in Nierumuzha. Especially during the soft dough stage, they had higher expression in purple-seedcoat hulless barley than in white-seedcoat hulless barley (*P* < 0.01; Fig. 9A-K). In contrast, *ANR* was significantly upregulated in Kunlun10 as compared to Nierumuzha (*P* < 0.01; Fig. 9L). The above structural genes and TF may play important roles in the synthesis of anthocyanins in purple Tibetan hulless barley.

## Discussion

Fruits and vegetables containing anthocyanins are an important part of the human diet, and extracted anthocyanins can be added to foods not only to increase nutritional values, but also as natural preservatives (replacing synthetic preservatives such as benzoic acid) and coloring agents [34]. Thus, anthocyanins are considered natural, safe, and healthy food additives [35]. Anthocyanidins naturally occur in more than 250 plant species, representing 73 genera and 27 families, of which more than 20 different anthocyanins currently known, the six most common are the aglycones pelargonidin (Pg), cyanidin (Cy), delphinidin (Dp), peonidin (Pn), petunidin (Pt), and Malvidin (Mv) [11, 36]. These six anthocyanidins bound to sugars by glycosidic bonds to form different types of anthocyanins [7]. Here, we profiled the anthocyanin content and specific anthocyanin compounds of two varieties of Tibetan hulless barley with different colored seedcoats: purple (Nierumuzha) and green (Kunlun10). We then investigated the underlying genetic and molecular mechanisms driving the differences in color formation.

To our knowledge, fewer studies have formally characterized anthocyanin composition in Tibetan hulless barley. Using MRM, we identified 32 anthocyanin compounds corresponding to the six-common anthocyanin aglycones in the seedcoats of the two varieties. We also detected five proanthocyanidin compounds: Procyanidin A1, Procyanidin A2, Procyanidin A3, Procyanidin B2, and Procyanidin B3 (Table S1). Procyanidins are polyphenolic compounds that produce anthocyanins when heated in an acidic medium [37]. Here, we detected Procyanidin A1 and Procyanidin A2 only in Nierumuzha during the middle and late stages of color formation (late milk and soft dough), suggesting that these two procyanidins became colored anthocyanidins during the later stages of grain color formation. Four additional compounds, Pseudopurpurin, Rhein, Taxifolin, and Gentisin, were predicted to have similar structures and molecular formulas to flavonoids and anthocyanins, but it was unclear whether these compounds were truly anthocyanins and, if so, to which class of anthocyanins they belonged (Table S1). In a previous study, delphinidin 3-glucoside, cyanidin 3-glucoside, petunidin 3-glucoside, and nine unidentified anthocyanins were identified in a pelted purple barley (CI-1248), the most abundant of which were cyanidin 3-glucoside, delphinidin 3-glucoside, and an unidentified anthocyanin compound [6]. In contrast, we found that the three most abundant anthocyanins in Nierumuzha were cyanidin O-malonyl-malonylhexoside, cyanidin O-rutinoside, and peonidin O-hexoside. This discrepancy may have been due to the differences between the two purple varieties investigated: the purple variety examined by Bellido et al. was CI-1248 (i.e., with an attached glume) [6], while Nierumuzha is a naked/hulless barley variety (i.e., without an attached glume) [2, 3]. The inclusion of the glume in the previous study may have led to the seeming differences in anthocyanin composition and content between our work and that of Bellido et al. (2009) [6]. Notably, anthocyanin composition and content differed significantly between Nierumuzha and Kunlun10: 15 more anthocyanin compounds were identified in Nierumuzha, and the anthocyanin content of Nierumuzha was 25 times greater than that of Kunlun10. These differences may explain the differences in seedcoat color between the two varieties.

Anthocyanins are responsible for the purple coloration of wheat-crop grains, and variations in anthocyanin content have been linked to the differential expression of key genes encoding structural enzymes in the anthocyanin biosynthesis pathways [38, 39]. Although several hulless barley genomes are available, no transcriptomic studies investigating color synthesis in purple grains have been performed using them [3, 40]. To identify key structural genes modulating the differential pigmentation of the seedcoats of Nierumuzha and Kunlun10, we de novo sequenced and assembled the Nierumuzha and Kunlun10 transcriptomes from seedcoat samples collected at three stages of color development. Based on these transcriptomes, we identified 19 DEGs related to proanthocyanin-anthocyanin biosynthesis, 16 of which included a total of 61 putative SNPs. These genes have been variously classified as early anthocyanin-biosynthesis genes (e.g., *PAL, C4H,* and *4CL*), middle anthocyanin-biosynthesis genes (e.g., *CHS, CHI, F3H,* and *F3’H*), and late anthocyanin-biosynthesis genes (*DFR, ANS,* and *GT*) [41].

Four genes associated with early anthocyanin biosynthesis (three *PAL* and one *4CL*) were significantly upregulated in Nierumuzha as compared to Kunlun10, suggesting that the expression of these genes leads to anthocyanin accumulation in the seedcoat and the initial development of the purple color in Nierumuzha during the early milk stage. Wan et al. analyzed the results of Zhonghua16 in pink seedcoat, and the results showed that the expression of *PAL* and *4CL* increased with the growth of peanut [42]. RsCHS is up-regulated in the formation of fleshy roots in red radish and is an important gene for its red color formation [43]. Our results also showed that the *C4H* gene was the first key regulator of purple formation in the seedcoat of Nierumuzha: the significant upregulation of *C4H* in Nierumuzha as compared to Kunlun10 suggested that this molecule acts as a substrate for anthocyanin synthesis and accumulation.

Nine genes associated with middle anthocyanin biosynthesis (two *CHS*, two *CHI*, two *F3H*, one *F3’H* and two *DFR*) were significantly upregulated in Nierumuzha as compared to Kunlun10, suggesting that these genes encode products that increase anthocyanin accumulation in the seed coat. Notably, *F3H* was significantly higher than that in Kunlun10, providing middle products for the accumulation of grain coats in the purple variety, which was the main reason for the middle differentiation of grain coat. It was worth mentioning that the expression of *F3H* also significant up-regulation in late milk (HORVU2Hr1G110130) and soft dough (novel.5509) stage of Kunlun 10, which might be because most *F3H* genes play catalytic roles in other branches of the flavonoid pathway [44]. One *DFR* gene (HORVU7Hr1G093480) was not expressed in Kunlun10 during any of the critical color-development periods examined (early milk, late milk, and soft dough). However, this gene was expressed in all three developmental stages in Nierumuzha, with significant increases in expression level between consecutive stages. This significant upregulation of *DFR* may have facilitated phycocyanin accumulation. Consistent with this, the introduction of *DFR* into a white petunia led to the expression of a brick-red phenotype [45]. The second *DFR* gene (HORVU3Hr1G056560) was significantly downregulated in the soft dough stage in both varieties as compared to the early milk stage. However, expression level in the soft dough stage as compared to the early milk stage decreased by 95.85% in Kunlun10 but only by 14.26% in Nierumuzha. This might be because DFR catalyzes three dihydroflavonols (dihydrokaempferol, dihydroquercetin, and dihydromyricetin) and produces the corresponding colorless proanthocyanidin sapogenins (pro-geranidin sapogenins, pro-cornflower sapogenins, and pro-cyanidin sapogenins) [45]. Thus, *DFR* might be the first key gene affecting anthocyanin synthesis during the late milk stage.

During the late milk and soft dough stage, the *ANR* gene was significantly upregulated in Kunlun10 as compared to Nierumuzha, providing products for absence of color in the white variety. In contrast, *ANS, GT,* and *ACT* were significantly upregulated in Nierumuzha as compared to Kunlun10, providing products for the accumulation of anthocyanins in the seedcoat of the purple variety. These differential expression patterns may underlie the difference in seedcoat color during the late stage of color development. It has been shown that *ANR* catalyzes the reduction of anthocyanin aglylate and the corresponding synthesis of epicatechin, epofodouin, and epigallocatechin [46]. In addition, the overexpression of the *ANR* gene from *Theobroma cacao* in transgenic *A. thaliana* reduced the content of anthocyanin glycosides in the hypocotyl of the mutant [47]. Finally, the overexpression of *ANS* in *Perilla frutescens* increased the conversion of proanthocyanidin glycosides to anthocyanidin glycosides in an HCl-acidified environment [48]. These results suggested that there might be a competitive relationship between *ANR* and *ANS*. Importantly, we found that almost all genes associated with anthocyanin biosynthesis tended to be upregulated in the later stages of anthocyanin glycoside synthesis as compared to the earlier stages. This pattern of upregulation leads to a series of complex catalytic reactions that color the green seedcoats white or purple. In addition, a nonsense mutation was found at 1195 bp in the CDS of the *ANS* of Kunlun10, resulting in premature termination of protein translation. We then searched the *ANS* of two white barley varieties, Morex and Golden promise, using Gramene (https://www.gramene.org/) and also found T at the position of 1195 bp. Zhuang et al.identified a putative nonsense mutation in the coding sequence of the *DFR* gene could lead to a nonfunctional protein in the green turnip, which may impair the accumulation of anthocyanins in green turnip skin [32]. The mutation of this study might encode a nonfunctional protein, further reducing anthocyanin accumulation in Kunlun10.

Interestingly, among the 4186 DEGs, we discovered a gene with no functional annotation (HORVU7Hr1G116630) that was consistently upregulated in Nierumuzha (FPKM: 954.8–3968.18) as compared to Kunlun10 (FPKM: 0.74–1.51), but was gradually downregulated in both varieties between the early milk and soft dough stages. These results suggested that this unannotated gene might be involved in the early stages of anthocyanin synthesis. In addition, this gene might be positively regulated by *homeobox* (*HB*) TFs and negatively regulated by *lateral organ boundaries domain* (*LOB*) TFs and/or other TFs (Fig. 4). Although we identified no SNPs in the CDS of this gene between the two varieties, there was a SNP in the upstream promoter region. This might imply that the differential regulation of the promoter may play a critical role in determining the expression patterns of the gene. Further examination of this gene is needed, because there is a highly significant positive correlation between the expression of this gene and anthocyanin content.

The activities of the structural genes in the proanthocyanidin-anthocyanin biosynthetic pathways are regulated by TFs in the MYB, bHLH, and WD40 families, which form the ternary complex MBW [49]. This result suggested that several members of *MYB*, *bHLH*, and *WD40* families were the main drivers of transcriptional differences between the two Tibetan hulless barleys (Fig. 4C). Many *bZIPs* were also differentially expressed between the two varieties, suggesting that *bZIP* TFs might also play key roles in anthocyanin biosynthesis (Fig. 3). Consistent with this, several previous studies have shown that *bZIP* TFs play an important role in plant coloration. For example, overexpression of the *bZIP* transcription factor *MdHY5* in apple promoted anthocyanin accumulation by regulating the expression of downstream anthocyanin biosynthesis genes [50]. In addition, Liu et al. demonstrated that *PybZIPa* played a role in light-induced anthocyanin accumulation by binding to tandem G-boxes in the *PyUFGT* promoter, leading to PyUFGT activation [51]. Finally, screening of 16 DEGs associated with phytohormone signaling, including four genes that downregulated AUX signaling, suggested that hormones had an antagonistic effect on pigment formation [41]. Consistent with this, we identified one gene regulated by *AUX/IAA* (HORVU3Hr1G022540) that was upregulated in Kunlun10 and downregulated in Nierumuzha. Several TFs not previously implicated in the proanthocyanidin-anthocyanin biosynthetic pathways were significantly correlated with anthocyanin synthesis: *HB* (HORVU4Hr1G083750), *C2H2* (HORVU3Hr1G086690), and an unknown TF (nov. 4878) were positively correlated, while *CSD* (HORVU6Hr1G012510) and *MADS-MIKC* (HORVU6Hr1G032220) were negatively correlated. The identification of these TFs provides a basis for future functional characterizations of seedcoat pigmentation in barley.

## Materials and methods

### Plant materials

The purple Tibetan hulless barley cultivar (Nierumuzha) and the green cultivar (Kunlun 10) were obtained from the Academy of Agriculture and Forestry Sciences of Qinghai University, Xining, China. In April 2020, the seeds were planted in the experimental field of the Academy of Agricultural and Forestry Sciences. Seeds of both varieties were collected at the early milk, late milk, and soft dough stages (based on Zadoks growth scale: Z73–Z85, early milk to soft dough) [52]. Nine spikes were collected per variety per stage, representing biological replicates: three spikes were used to determine total anthocyanins, and the remaining six were used for transcriptomic, and metabolomic sequencing of the seedcoat. Three biological replicates were taken for each developmental stage. Then the sampled grains and seedcoats were immediately frozen in liquid nitrogen and stored in a freezer at − 80 °C.

### Total anthocyanin quantification

A spectrophotometric pH differential method was used to estimate the total anthocyanins in the barley grains following Yuan et al. [53]. Three biological replicates were used for each variety at each stage. One-way analyses of variance (ANOVAs) in SPSS 20.0 (SPSS Inc., USA) were used to identify statistically significant differences among mean total anthocyanin contents [31].

### Anthocyanin extraction and MRM

MRM abalysis was performed by Novogene (Beijing, China). Freeze-dried seedcoat samples (0.1 mg) were separately ground in liquid nitrogen. Next, protein precipitation was performed by adding 1 mL of 80% methanol (volume ratio of methanol:water:formic acid, 4:1:0.001) to each ground sample and allowing the mixture to cool in an ice bath for 5 min. Next, samples were centrifuged at 15,000 × *g* for 15 min at 4 °C and the supernatants were collected. Each supernatant was filtered through a 0.22-μm membrane, and the filtrate was diluted from 80% methanol to 60% methanol. The resulting extracts were analyzed using the liquid chromatography mass spectrometry (LC–MS) system (SCIEX QTRAP 6500 +), with the following analytical conditions: high-performance liquid chromatography (HPLC) column, waters ACQUITY UPLC HSS T3 (2.1 mm × 100 mm); solvent system, water (A: 0.1% formic acid, B: acetonitrile); gradient program, 80% A: 20% B at 0 min and 1 min, 25% A: 75% B at 3 min and 4 min, 80% A: 20% B at 4.5 min, 6.5 min, and 7.5 min; flow rate, 0.20 mL/min; temperature, 40 °C; injection volume, 2 μL. The MS conditions were as follows: curtain gas, 35; collision gas, medium; ion spray voltage, − 4500; temperature, 500 °C; ion source gas 1:50; ion source gas 2:50. An equal volume of each experimental sample was mixed to generate the quality-control sample. After obtaining the signal intensity (CPS) of the characteristic ions, the MS results for each sample were analyzed using SCIEX OS V1.4: peaks were integrated and calibrated, such that each peak area was proportional to the relative abundance of the corresponding substance. Three biological replicates were taken per developmental stage for MRM analyses.

### RNA-seq analysis

Total RNA was extracted from seedcoats of the Nierumuzha and Kunlun10 varieties at each developmental stage using MiniBEST Universal RNA Extraction Kit (TaKaRa, Tokyo, Japan); three biological replicates were analyzed per variety per stage (*n* = 18 in total). RNA-seq and assembly were performed by Novogene (Beijing, China). Sequencing libraries were generated using the RNA Nano 6000 Assay Kit and the Bioanalyzer 2100 system (Agilent Technologies, Santa Clara, CA, USA), following the manufacturer’s instructions. The library preparations were sequenced on an Illumina HiSeq 2500 platform, and paired-end reads were generated. Raw reads were cleaned by removing adaptor sequences and low-quality sequence reads from the data sets. Clean reads were then mapped to the barley reference genome sequence (ftp://ftp.gramene.org/pub/gramene/release-63/fasta/hordeum_vulgare/dna/).

We identified DEGs between the two varieties at each developmental stage based on FPKM values using the DEGseq R package [54]. *P*-values were adjusted using q-values [55]. We considered DEGs with the adjusted *P*-value < 0.05 and |log2 (fold change)|≥ 1 significantly differentially expressed [31]. DEG functions were annotated against the following databases: Swiss-Prot (manually annotated and reviewed protein sequences; http://www.expasy.ch/sprot), Pfam (protein families; http://pfam.xfam.org/), KEGG (Kyoto Encyclopedia of Genes and Genomes; www.kegg.jp/kegg/kegg1.html) and GO (Gene Ontology; http://wego.genomics.org.cn/cgi-bin/wego/index.pl). Correlation analysis was performed using the OmicStudio tools (https://www.omicstudio.cn/tool/62).

GATK2 (v3.7) was used to perform SNP calling [56]. Raw vcf files were filtered with the standard GATK filter method and other parameters were set as follows: cluster, 3; WindowSize, 35; QD < 2.0; FS > 30.0; DP < 10 [57]. WGCNA was performed using the R package WGCNA 1.51 [58]. WGCNA is a set of functions used to calculate various weighted association analyses and can be used for network construction, gene screening, gene cluster identification, topological feature calculation, data simulation, and visualization [58]. After ranking the genes from largest to smallest based on the median absolute deviation (and eliminating all genes with FPKM values of 0 at any stage in either variety), we selected the top 25,375 genes for WGCNA analysis. To obtain an appropriate scale-free topology, we chose a power of eight following the scale-free topology model [59]. A scale-free network was then constructed by choosing the weight value β = 10, and then the modules were divided according to the hybrid dynamic shear criterion, followed by calculating the eigenvectors (eigengenes) of each module in turn and merging the closer modules to obtain 25 co-expression modules corresponding to gene clusters. The intramodular connectivity (*K*_*IM*_) was calculated to measure the connection strength between a given gene and other genes in a given modul [60]. The five genes with the highest linkage values across the six significantly correlated modules, as well as the genes associated with anthocyanin synthesis, were selected as hub genes and their interaction networks were visualized using Cytoscape [59]. The interactions in the STRING (https://cn.string-db.org/) protein interactions database were applied to analyze the differential gene protein interactions network.

### RT-PCR and qRT-PCR analysis

Total RNA was extracted from seedcoats of Nierumuzha and Kunlun10 using TaKaRa MiniBEST Universal RNA Extraction Kit (Takara, Tokyo, Japan), and cDNA was synthesized using a PrimeScriptTM RT reagent kit with gDNA Eraser (Takara, Tokyo, Japan) following to the manufacturer’s instructions. To reduce individual differences, RNA samples from each replicate were mixed in equal quantities to form pooled samples representing each variety at each developmental stage [31]. The RT-PCR amplification system and procedure were referred to the method of Yao et al. [61]. qRT-PCRs were performed using the SYBR Premix Ex Taq II Kit (Takara, Tokyo, Japan) on a Roche LightCycler480 II (Roche Molecular Biochemicals, Mannheim, Germany). Each reaction volume (10 μl) contained 5.0 μl of 2 × SYBR Premix Ex Taq, 0.4 μl of each primer, 1.0 μl of cDNA, and 3.2 μl of ddH_2_O. Primers for the 12 genes associated with anthocyanin biosynthesis (11 structural genes and one transcription factor gene) were designed using Primer 7.0 (Table S6). The cycling parameters were as follows: 95 °C for 30 s; 95 °C for 5 s, 60 °C for 30 s (40 cycles); 95 °C for 5 s, 60 °C for 1 min (1 cycle); and 50 °C for 30 s (1 cycle). *TC139057* was selected as an internal standard for expression analysis [62]. The expression levels of all 12 genes were determined simultaneously. Gene expression levels were calculated relative to that of *TC139057* using the 2^–ΔΔCt^ method [63]. Each RT-qRCR analysis was performed three times in parallel [31].

## Conclusions

Collectively, the mechanism of anthocyanin accumulation in Tibetan hulless barley seedcoat was analyzed by using metabolomic and transcriptomic analyses and RT-qPCR. Our results revealed that a series of significantly different 39 anthocyanin compounds, 19 structural genes and 22 TFs were obtained between purple and white Tibetan hulless barley seedcoats. Then we found *PAL, C4H, 4CL, CHS, CHI, F3H, F3’H, DFR, ANS, GT,* and *ACT,* were significantly upregulated in Nierumuzha as compared to Kunlun10, resulting in notably greater levels of 15 anthocyanins compounds in Nierumuzha, while *ANR* was significantly downregulated in Nierumuzha as compared to Kunlun10, resulting in lower contents of three anthocyanins compounds in Nierumuzha. Furthermore, we found 61 putative SNPs of 13 structural genes, especially a nonsense mutation was identified in the coding sequence of the *ANS* gene in Kunlun10. The above may be the main reason for the difference in seedcoat color between the two Tibetan hulless barley cultivars. In conclusion, we constructed the proanthocyanin-anthocyanin biosynthesis pathway of Tibetan hulless barley. A series of compounds, structural genes and TFs responsible for the differences between purple and white hulless barley seeds were obtained in this pathway. These results improve our understanding of the molecular mechanisms of anthocyanin accumulation and biosynthesis in barley seeds and provide a series of candidate genes that can be used to breed anthocyanin-rich cultivars.

## Supplementary Information

**Additional file 1: Figure S1.** Expression heatmap of 40 DEGs related to anthocyanin synthesis. Heatmap shows the patterns of differential expression across the 40 DEGs at each stage. Cell colors correspond to the log10 magnitude of the difference in expression level [log10(fold change values + 1)]: redder cells indicate upregulation, and bluer cells indicate downregulation. **Figure S2. **Gene cluster dendrograms and modules. (A) Gene cluster dendrograms based on TOM-based dissimilarity. (B) Module divisions after dynamic tree cutting. (C) Module divisions after merging similar modules. Different colors indicate different modules.

**Additional file 2: Table S1.** Abundance of Anthocyanin Compounds in the Two Hulless Barley Varieties, Nierumuzha (PC1–PC3) and Kunlun10 (WC1–WC3), at Three Stages of Seed Development.

**Additional file 3: Table S2.** Statistics for the Transcriptome Sequencing Dataset, Including Quality Checking and Comparison with the Barley Reference Genome.

**Additional file 4: Table S3.** 134 DEGs related to anthocyanin synthesis.

**Additional file 5: Table S4.** SNPs Comparison of the Candidate Genes Between the Two Tibetan Hulless Barleys.

**Additional file 6: Table S5.** Prediction of protein-protein interactions (PPIs).

**Additional file 7: Table S6.** Primer sequences used in the present study.

## Data Availability

The datasets generated and analyzed during the current study are available in the [National Center for Biotechnology Information (NCBI). The datasets generated for this study can be found in the Sequence Read Archive (SRA) accession number: SRR18355550, SRR18355551, SRR18355552, SRR18355553, SRR18355554, SRR18355555, SRR18355556, SRR18355557, SRR18355558, SRR18355559, SRR18355560, SRR18355561, SRR18355562, SRR18355563, SRR18355564, SRR18355565, SRR18355566, SRR18355567.

## Abbreviations

DEGs: Differentially Expressed Genes
CHI: Chalcone Isomerase
CHS: Chalcone Synthase
F3H: Dihydroflavonol-3-Hydrogenase
F3’H: Dihydroflavonol-3’-Hydrogenase
F3′5’H: Dihydroflavonol-3’-5’-Hydrogenase
DFR: Dioxanonol-4-Reductase
ANS: Anthocyanidin Synthase
bHLH: Basic Helix-Loop-Helix
MYB: V-Myb Avian Myeloblastosis Viral Oncogene Homolog
PAL: Phenylalanine Ammonia-Lyase
MRM: Multiple Reaction Monitoring
LC–MS: Liquid Chromatography Mass Spectrometry
HPLC: High-Performance Liquid Chromatography
SE: Standard Error
FPKM: Fragments Per Kilobase Per Million
TFs: Transcription Factors
WGCNA: Weighted Correlation Network Analysis
PPIs: Protein–Protein Interactions
