# Supplementary material for: Accumulation and regulation of anthocyanins in white and purple Tibetan Hulless Barley (Hordeum vulgare L. var. nudum Hook. f.) revealed by combined de novo transcriptomics and metabolomics
Source: BMC Plant Biol. 2022 Aug 4;22:391. doi: 10.1186/s12870-022-03699-2 (PMC9351122; doi:10.1186/s12870-022-03699-2)
Supplement: Supplementary file 1 — Additional file 1: Figure S1. Expression heatmap of 40 DEGs related to anthocyanin synthesis. Heatmap shows the patterns of differential expression across the 40 DEGs at each stage. Cell colors correspond to the log10 magnitude of the difference in expression level [log10(fold change values + 1)]: redder cells indicate upregulation, and bluer cells indicate downregulation. Figure S2. Gene cluster dendrograms and modules. (A) Gene cluster dendrograms based on TOM-based dissimilarity. (B) Module divisions after dynamic tree cutting. (C) Module divisions after merging similar modules. Different colors indicate different modules. [file 12870_2022_3699_MOESM1_ESM.docx]

**Supplementary Figures**

**
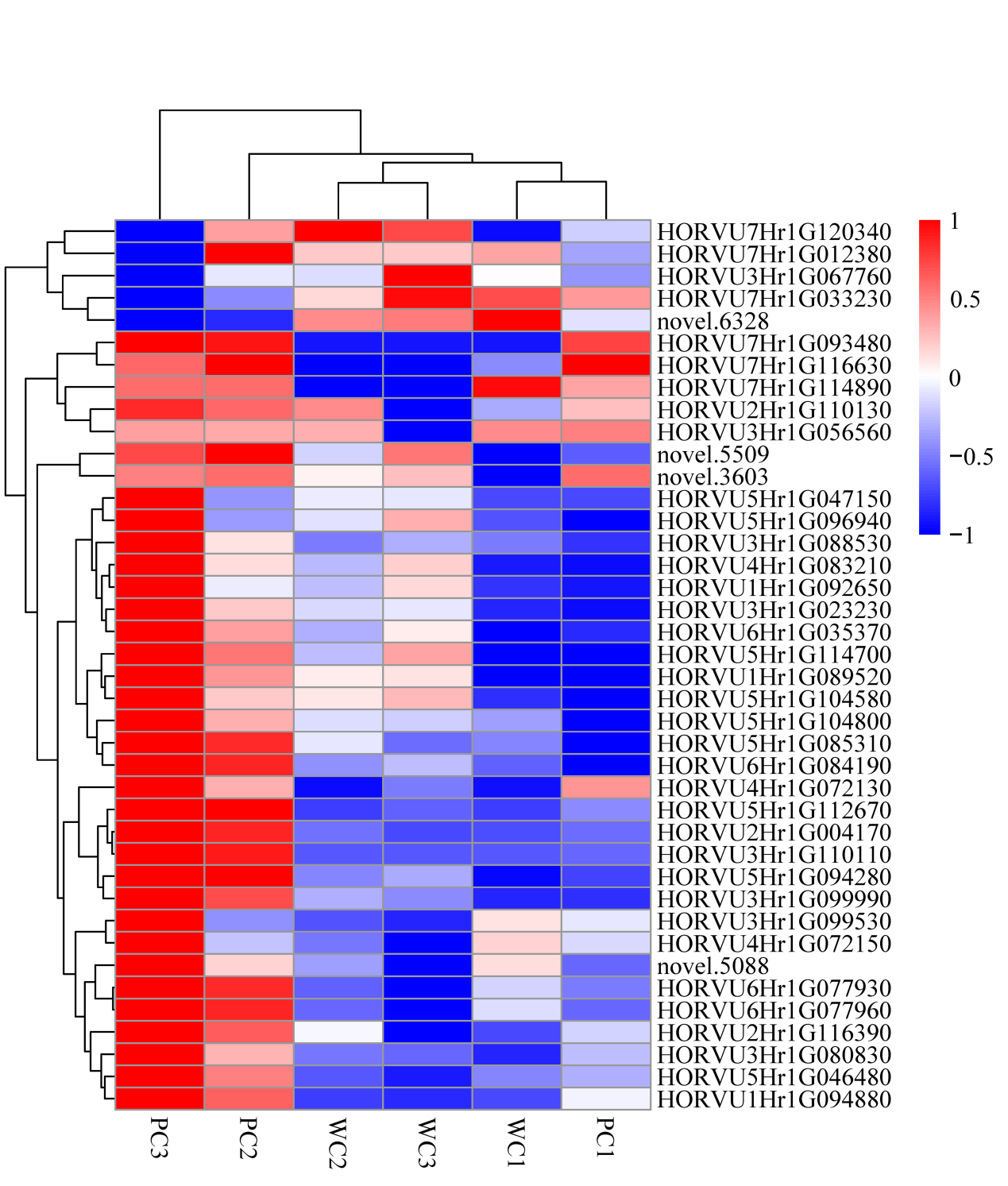
**

**Additional file 1: Figure S1.** Expression heatmap of 40 DEGs related to anthocyanin synthesis. Heatmap shows the patterns of differential expression across the 40 DEGs at each stage. Cell colors correspond to the log10 magnitude of the difference in expression level [log10(fold change values + 1)]: redder cells indicate upregulation, and bluer cells indicate downregulation.


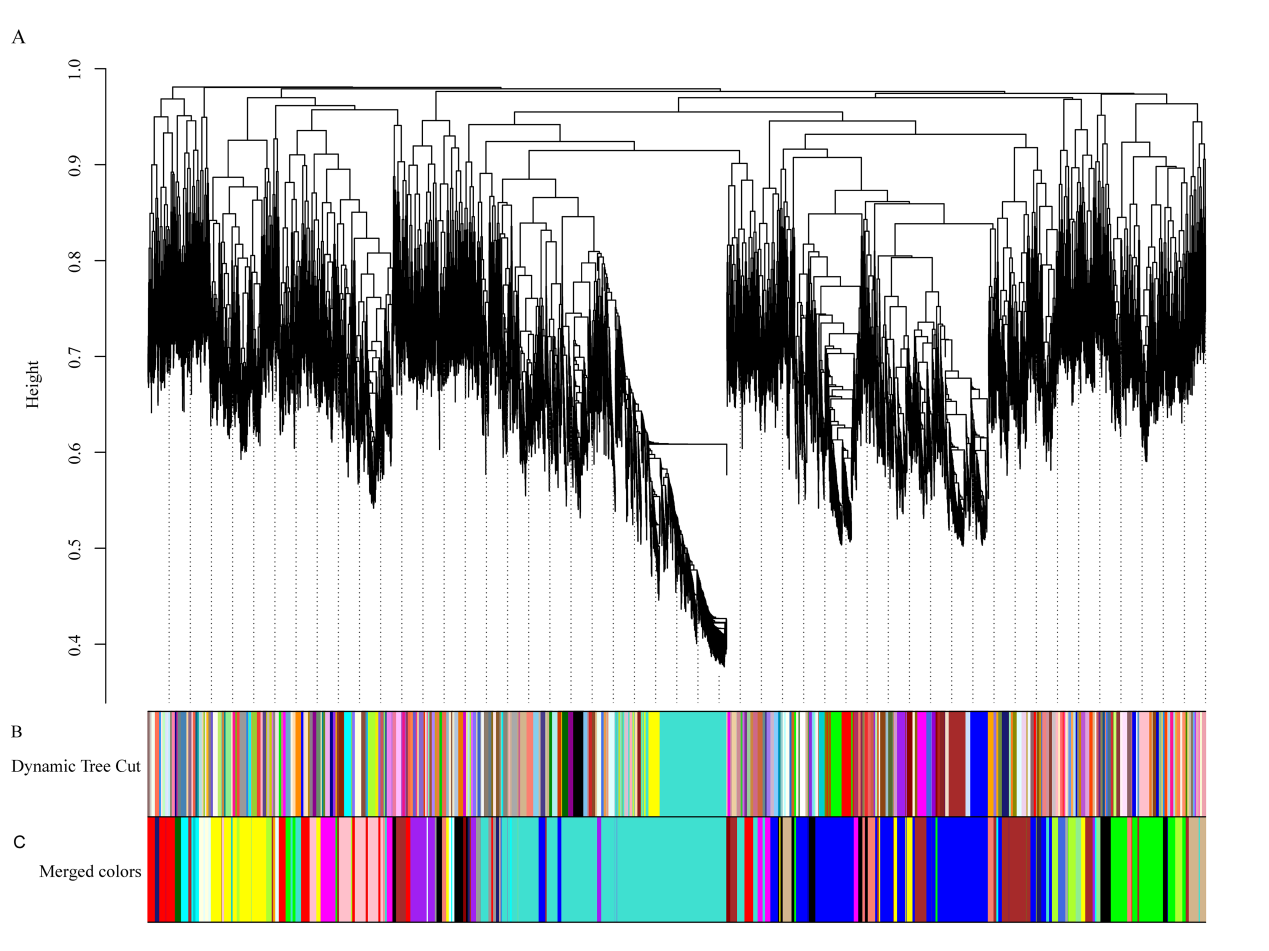


**Additional file 2: Figure S2.** Gene cluster dendrograms and modules. (A) Gene cluster dendrograms based on TOM-based dissimilarity. (B) Module divisions after dynamic tree cutting. (C) Module divisions after merging similar modules. Different colors indicate different modules.
