# Supplementary material for: Accumulation and regulation of anthocyanins in white and purple Tibetan Hulless Barley (Hordeum vulgare L. var. nudum Hook. f.) revealed by combined de novo transcriptomics and metabolomics
Source: BMC Plant Biol. 2022 Aug 4;22:391. doi: 10.1186/s12870-022-03699-2 (PMC9351122; doi:10.1186/s12870-022-03699-2)
Supplement: Supplementary file 2 — Additional file 2: Table S1. Abundance of Anthocyanin Compounds in the Two Hulless Barley Varieties, Nierumuzha (PC1–PC3) and Kunlun10 (WC1–WC3), at Three Stages of Seed Development. [file 12870_2022_3699_MOESM2_ESM.docx]

**Table S1. Abundance of Anthocyanin Compounds in the Two Hulless Barley Varieties, Nierumuzha (PC1–PC3) and Kunlun10 (WC1–WC3), at Three Stages of Seed Development**

| Compounds | MW  (Da) | CPS | | | | | | | | | | | |
| --- | --- | --- | --- | --- | --- | --- | --- | --- | --- | --- | --- | --- | --- |
|  |  | WC1 | SE | WC2 | SE | WC3 | SE | PC1 | SE | PC2 | SE | PC3 | SE |
| Oenin chloride | 509.1 | 14763 | 1081.87 | 8243 | 1818.09 | 10304 | 538.57 | 6434 | 1228.90 | 8040 | 1057.66 | 26833 | 4867.86 |
| Myrtillin chloride | 481.1 | 8927 | 1294.13 | 12263 | 601.78 | 14853 | 1708.11 | 6877 | 345.66 | 18553 | 1233.14 | 12760 | 2125.86 |
| Keracyanin chloride | 611.1 | 9782 | 1928.17 | 12997 | 1009.47 | 5397 | 271.93 | 13283 | 1667.94 | 12880 | 670.00 | 7749 | 1193.30 |
| Pelargonin chloride | 611.1 | 19870 | 12568.88 | 44720 | 17239.49 | 74877 | 14051.89 | 3763 | 542.19 | 64883 | 7381.77 | 36577 | 7104.48 |
| Delphinidin O-malonylhexoside | 551.1 | 2644 | 605.51 | 4771 | 418.68 | 9030 | 441.27 | 3770 | 416.26 | 233867 | 28466.88 | 469600 | 61932.95 |
| Pelargonidin 3-O-malonylhexoside | 519.1 | 0 | 0.00 | 0 | 0.00 | 0 | 0.00 | 0 | 0.00 | 3545 | 322.26 | 19227 | 2725.81 |
| Rosinidin O-hexoside | 477.1 | 21675 | 2638.55 | 15030 | 1744.59 | 34723 | 7428.60 | 28317 | 4727.12 | 28437 | 3439.13 | 87630 | 8705.04 |
| Malvidin 3,5-diglucoside | 655.2 | 338533 | 37105.44 | 77587 | 23791.80 | 60330 | 8123.49 | 352733 | 29350.53 | 35433 | 5118.49 | 93977 | 14924.99 |
| Cyanidin 3-O-glucosyl-malonylglucoside | 697.1 | 47603 | 5891.01 | 188400 | 27139.82 | 190167 | 14350.03 | 37560 | 7115.53 | 160500 | 32379.62 | 186133 | 40590.80 |
| Peonidin O-hexoside | 463.1 | 303933 | 158784.58 | 244133 | 45601.79 | 753033 | 89718.02 | 184400 | 12474.37 | 7107667 | 193184.71 | 14310000 | 2945555.97 |
| Pelargonidin 3-O-malonyl-malonylhexoside | 605.1 | 60060 | 2575.01 | 127767 | 5739.63 | 141567 | 14867.53 | 67780 | 2864.07 | 85240 | 6760.87 | 146333 | 17118.51 |
| Malvidin O-hexoside | 493.1 | 0 | 0.00 | 0 | 0.00 | 0 | 0.00 | 0 | 0.00 | 21541 | 2469.97 | 157420 | 45917.24 |
| Procyanidin A2 | 577.0 | 0 | 0.00 | 0 | 0.00 | 0 | 0.00 | 0 | 0.00 | 19841333 | 8870054.42 | 94703333 | 7636375.67 |
| Cyanidin O-malonyl-malonylhexoside | 621.1 | 3642667 | 977849.85 | 1945700 | 485707.39 | 986733 | 109378.72 | 2232000 | 1626477.17 | 39896667 | 4101881.68 | 62720000 | 6521909.23 |
| Delphinidin O-malonyl-malonylhexoside | 637.1 | 0 | 0.00 | 5660 | 1299.43 | 9652 | 1137.92 | 0 | 0.00 | 2764667 | 388439.61 | 2259000 | 219642.89 |
| Peonidin chloride | 317.1 | 4439 | 633.86 | 1361 | 47.63 | 554 | 101.89 | 2237 | 58.05 | 528800 | 51651.52 | 529700 | 35339.92 |
| Cyanidin chloride | 303.1 | 6786 | 805.07 | 7774 | 901.00 | 6313 | 927.35 | 4723 | 381.75 | 59743 | 5868.21 | 165400 | 21258.65 |
| Idaein chloride | 465.1 | 9391 | 459.35 | 13843 | 808.35 | 16757 | 2728.96 | 5036 | 607.08 | 642933 | 80081.98 | 1029333 | 123637.91 |
| Ferulylpelargonidin di-O-hexosyl-O-pentoside | 919.0 | 1129 | 51.96 | 6086 | 397.41 | 10259 | 892.72 | 0 | 0.00 | 6836 | 1077.31 | 17047 | 3145.22 |
| Procyanidin A1 | 577.0 | 0 | 0.00 | 0 | 0.00 | 0 | 0.00 | 0 | 0.00 | 17367333 | 8773731.32 | 86973333 | 6914017.16 |
| Pseudopurpurin | 332.9 | 1492 | 396.49 | 6741 | 1066.32 | 5008 | 525.52 | 0 | 0.00 | 3866 | 274.46 | 2595 | 166.23 |
| Petunidin 3-O-rutinoside | 625.3 | 37543 | 3599.69 | 31210 | 15173.24 | 37770 | 8254.17 | 32123 | 9307.57 | 39653 | 6297.82 | 31560 | 1629.08 |
| Cyanidin O-rutinoside | 595.2 | 17616667 | 2065244.13 | 19041333 | 2776372.69 | 14976667 | 1935777.19 | 10178667 | 1558135.21 | 13060000 | 1935019.38 | 4676667 | 668036.18 |
| Cyanidin O-syringic acid | 465.1 | 7909 | 1233.47 | 11190 | 228.69 | 15527 | 662.75 | 3835 | 272.33 | 462567 | 24857.66 | 895100 | 62961.26 |
| Cyanidin O-acetylhexoside | 489.1 | 16961 | 2222.03 | 3030 | 289.76 | 4674 | 891.31 | 8427 | 1279.81 | 50300 | 2766.57 | 166067 | 20071.46 |
| Delphinidin chloride | 319.0 | 3269 | 687.92 | 3568 | 325.55 | 4178 | 88.82 | 3191 | 680.69 | 3337 | 205.51 | 3782 | 305.23 |
| Procyanidin A3 | 577.1 | 309700 | 33029.23 | 190733 | 32500.05 | 140033 | 13387.43 | 325433 | 54106.96 | 124000 | 13284.20 | 64763 | 1848.41 |
| Procyanidin B2 | 577.1 | 16420000 | 1126232.66 | 9699333 | 684953.53 | 7873333 | 459853.60 | 16750000 | 1260476.10 | 10963333 | 1252251.31 | 4121000 | 193517.44 |
| Procyanidin B3 | 577.1 | 12702000 | 2887790.16 | 7863333 | 807501.29 | 6536000 | 920195.09 | 14174333 | 938693.95 | 7778000 | 1140470.08 | 3396333 | 128515.89 |
| Delphinidin 3-sophoroside-5-rhamnoside | 773.0 | 979 | 154.02 | 2299 | 259.62 | 4543 | 685.67 | 748 | 112.32 | 4005 | 791.82 | 6460 | 755.57 |
| Cyanidin 3-O-glucoside | 465.1 | 4018 | 716.27 | 3357 | 1143.82 | 4601 | 999.04 | 2361 | 503.19 | 235133 | 13134.05 | 362800 | 35548.98 |
| Petunidin-3-O-glucoside chloride | 495.1 | 1582 | 301.51 | 1468 | 293.92 | 1376 | 140.76 | 820 | 28.12 | 840 | 77.55 | 1188 | 129.13 |
| Pelargonidin chloride | 287.0 | 3165 | 523.18 | 6203 | 1341.55 | 6940 | 632.67 | 3073 | 658.05 | 43943 | 8851.19 | 212600 | 9182.05 |
| Cyanin chloride | 627.1 | 5674 | 802.07 | 6257 | 1040.71 | 7295 | 352.66 | 5569 | 857.47 | 24517 | 3950.40 | 130567 | 9386.34 |
| Callistephin chloride | 449.1 | 312733 | 57574.33 | 262200 | 13501.11 | 188200 | 16859.72 | 128200 | 3360.06 | 281067 | 43102.24 | 216633 | 4104.06 |
| Rhein | 316.9 | 3605 | 667.97 | 1747 | 321.32 | 4708 | 959.35 | 5129 | 423.20 | 2523 | 212.53 | 3598 | 396.06 |
| Pelargonidin O-acetylhexoside | 473.1 | 1048 | 262.97 | 4733 | 688.11 | 8833 | 902.36 | 1500 | 345.81 | 13595 | 588.10 | 87157 | 8916.19 |
| 3,3',4',5,5',7,8-Heptahydroxyflavone | 366.9 | 6295 | 814.90 | 7329 | 357.23 | 5752 | 215.27 | 8759 | 955.27 | 6232 | 477.10 | 5951 | 880.82 |
| Taxifolin | 336.9 | 7551 | 273.06 | 2870 | 603.38 | 976 | 12.15 | 6839 | 426.11 | 1807 | 360.01 | 0 | 0.00 |
| Gentisin | 290.9 | 5192 | 418.98 | 4484 | 997.35 | 7652 | 590.68 | 6305 | 1085.27 | 8353 | 360.49 | 10135 | 612.74 |
| Malvidin 3-galactoside chloride | 509.1 | 19303 | 486.86 | 15393 | 2761.96 | 21350 | 860.87 | 10042 | 1559.10 | 22540 | 3673.59 | 38943 | 4681.29 |

CPS. Count per second; SE. Standard error; MW. Molecular weight. Nierumuzha is a purple seedcoat cultivar; Kunlun10 is a white seedcoat cultivar; PC1–PC3. The early milk, late milk, and soft dough stages of Nierumuzha; WC1–WC3. The early milk, late milk, and soft dough stages of Kunlun10
