# Supplementary material for: Accumulation and regulation of anthocyanins in white and purple Tibetan Hulless Barley (Hordeum vulgare L. var. nudum Hook. f.) revealed by combined de novo transcriptomics and metabolomics
Source: BMC Plant Biol. 2022 Aug 4;22:391. doi: 10.1186/s12870-022-03699-2 (PMC9351122; doi:10.1186/s12870-022-03699-2)
Supplement: Supplementary file 3 — Additional file 3: Table S2. Statistics for the Transcriptome Sequencing Dataset, Including Quality Checking and Comparison with the Barley Reference Genome. [file 12870_2022_3699_MOESM3_ESM.docx]

**Table S2. Statistics for the Transcriptome Sequencing Dataset, Including Quality Checking and Comparison with the Barley Reference Genome**

| sample | raw reads | clean reads | clean bases (g) | error rate (%) | q20 (%) | q30 (%) | gc content (%) | total mapped | uniquely mapped | positive_map | negative_map |
| --- | --- | --- | --- | --- | --- | --- | --- | --- | --- | --- | --- |
| WC1_1 | 42560848 | 41324578 | 6.20 | 0.02 | 98.38 | 94.99 | 54.03 | 37898354 (91.71%) | 34001178 (82.28%) | 17005612 (41.15%) | 16995566 (41.13%) |
| WC1_2 | 40540728 | 39453484 | 5.92 | 0.03 | 98.02 | 94.13 | 53.03 | 35197105 (89.21%) | 31016313 (78.61%) | 15530488 (39.36%) | 15485825 (39.25%) |
| WC1_3 | 43694618 | 42326802 | 6.35 | 0.03 | 97.84 | 93.96 | 55.07 | 38937214 (91.99%) | 35003298 (82.7%) | 17493848 (41.33%) | 17509450 (41.37%) |
| WC2_1 | 44308308 | 42557468 | 6.38 | 0.02 | 98.15 | 94.57 | 53.77 | 37853341 (88.95%) | 33722449 (79.24%) | 16868371 (39.64%) | 16854078 (39.6%) |
| WC2_2 | 42645948 | 41435816 | 6.22 | 0.03 | 97.59 | 93.48 | 54.08 | 37023785 (89.35%) | 32865130 (79.32%) | 16443035 (39.68%) | 16422095 (39.63%) |
| WC2_3 | 44094550 | 42191966 | 6.33 | 0.02 | 98.04 | 94.26 | 53.56 | 37283578 (88.37%) | 32933404 (78.06%) | 16487379 (39.08%) | 16446025 (38.98%) |
| WC3_1 | 44976466 | 43368766 | 6.51 | 0.02 | 98.00 | 94.31 | 53.67 | 37451805 (86.36%) | 33153404 (76.45%) | 16575730 (38.22%) | 16577674 (38.22%) |
| WC3_2 | 43883926 | 42680360 | 6.40 | 0.03 | 97.45 | 93.20 | 53.79 | 37407879 (87.65%) | 33291960 (78.0%) | 16656306 (39.03%) | 16635654 (38.98%) |
| WC3_3 | 40709642 | 39472052 | 5.92 | 0.03 | 97.92 | 94.20 | 54.53 | 34741819 (88.02%) | 30867290 (78.2%) | 15444338 (39.13%) | 15422952 (39.07%) |
| PC1_1 | 44018888 | 42480032 | 6.37 | 0.03 | 97.54 | 93.34 | 54.33 | 38604852 (90.88%) | 34515009 (81.25%) | 17243262 (40.59%) | 17271747 (40.66%) |
| PC1_2 | 42720590 | 41211930 | 6.18 | 0.03 | 97.89 | 94.19 | 53.02 | 36943729 (89.64%) | 32467824 (78.78%) | 16235935 (39.4%) | 16231889 (39.39%) |
| PC1_3 | 41856596 | 40376392 | 6.06 | 0.02 | 98.04 | 94.43 | 54.32 | 36506083 (90.41%) | 32286134 (79.96%) | 16123870 (39.93%) | 16162264 (40.03%) |
| PC2_1 | 41606474 | 40095808 | 6.01 | 0.02 | 97.94 | 94.31 | 53.65 | 36021806 (89.84%) | 31924804 (79.62%) | 15929950 (39.73%) | 15994854 (39.89%) |
| PC2_2 | 44916396 | 42814680 | 6.42 | 0.02 | 98.00 | 94.38 | 54.40 | 38594978 (90.14%) | 33563187 (78.39%) | 16756961 (39.14%) | 16806226 (39.25%) |
| PC2_3 | 41145146 | 39346516 | 5.90 | 0.03 | 97.22 | 92.88 | 53.19 | 34447928 (87.55%) | 28821144 (73.25%) | 14392098 (36.58%) | 14429046 (36.67%) |
| PC3_1 | 43378774 | 42066910 | 6.31 | 0.03 | 97.55 | 93.45 | 54.32 | 35980895 (85.53%) | 31718418 (75.4%) | 15849262 (37.68%) | 15869156 (37.72%) |
| PC3_2 | 44364356 | 42544784 | 6.38 | 0.03 | 97.16 | 92.57 | 53.90 | 34971044 (82.2%) | 30845605 (72.5%) | 15425278 (36.26%) | 15420327 (36.24%) |
| PC3_3 | 46819098 | 45093154 | 6.76 | 0.03 | 97.52 | 93.5 | 53.23 | 39323397 (87.2%) | 33204400 (73.64%) | 16568915 (36.74%) | 16635485 (36.89%) |

PC1_1–PC1_3. Three biological repetitions of the Nierumuzha in early milk stage (PC1); PC2_1–PC2_3. Three biological repetitions of the Nierumuzha in late milk stage (PC2); PC3_1–PC3_3. Three biological repetitions of the Nierumuzha in soft dough stage (PC3); WC1_1–WC1_3. Three biological repetitions of the Kunlun10 in early milk stage (WC1); WC2_1–WC2_3. Three biological repetitions of the Kunlun10 in late milk stage (WC2); WC3_1–WC3_3. Three biological repetitions of the Kunlun10 in soft dough stage (WC3).
