# Supplementary material for: Accumulation and regulation of anthocyanins in white and purple Tibetan Hulless Barley (Hordeum vulgare L. var. nudum Hook. f.) revealed by combined de novo transcriptomics and metabolomics
Source: BMC Plant Biol. 2022 Aug 4;22:391. doi: 10.1186/s12870-022-03699-2 (PMC9351122; doi:10.1186/s12870-022-03699-2)
Supplement: Supplementary file 5 — Additional file 5: Table S4. SNPs Comparison of the Candidate Genes Between the Two Tibetan Hulless Barleys. [file 12870_2022_3699_MOESM5_ESM.docx]

**Table S4 SNPs Comparison of the Candidate Genes Between the Two Tibetan Hulless Barleys**

| Gene ID | Location | In the CDS or not | Nucleotide in reference genome | Nucleotide in Nierumuzha | Nucleotide in Kunlun10 | Amino acid in reference genomic | Amino acid in Nierumuzha | Amino acid in Kunlun10 |
| --- | --- | --- | --- | --- | --- | --- | --- | --- |
| HORVU1Hr1G022060 | 91840321 | Yes | T | C | T | S | G | S |
| HORVU2Hr1G089540 | 639286072 | No | G | A | G | / | / | / |
|  | 639285965 | No | G | T | G | / | / | / |
|  | 639286158 | No | T | C | T | / | / | / |
|  | 639288725 | No | G | A | G | / | / | / |
|  | 639436793 | No | C | G | C | / | / | / |
|  | 639436893 | No | A | G | A | / | / | / |
| HORVU5Hr1G112670 | 639992083 | No | C | A | C | / | / | / |
|  | 639992327 | Yes | A | G | A | T | T | T |
|  | 639992381 | Yes | T | C | T | C | C | C |
|  | 639992420 | Yes | T | C | T | A | A | A |
| HORVU5Hr1G046480 | 361451256 | Yes | A | G | A | I | V | I |
|  | 361451472 | Yes | G | A | G | C | Y | C |
|  | 361451484 | Yes | T | G | T | L | R | L |
| HORVU3Hr1G056560 | 423198950 | No | A | G | A | / | / | / |
|  | 423199606 | Yes | T | C | T | H | H | H |
| HORVU2Hr1G108250 | 713827024 | No | A | G | A | / | / | / |
|  | 713827216 | No | G | A | G | / | / | / |
| HORVU5Hr1G104580 | 619631546 | No | C | G | C | / | / | / |
|  | 619631754 | Yes | T | G | T | S | A | S |
|  | 619631784 | Yes | G | A | G | D | N | D |
|  | 619631924 | Yes | T | C | T | A | A | A |
|  | 619632048 | Yes | G | A | G | D | N | D |
| HORVU5Hr1G104800 | 619984535 | Yes | T | C | T | V | A | V |
|  | 619984583 | Yes | G | A | G | G | D | G |
|  | 619984870 | Yes | A | G | A | I | V | I |
| HORVU3Hr1G110110 | 679939889 | No | A | G | A | / | / | / |
|  | 679939928 | No | T | A | T | / | / | / |
|  | 679957208 | No | C | G | C | / | / | / |
|  | 679957319 | No | A | T | A | / | / | / |
|  | 679957359 | No | T | C | T | / | / | / |
|  | 680008379 | No | A | G | A | / | / | / |
|  | 680008412 | No | G | A | G | / | / | / |
|  | 680008417 | No | T | A | T | / | / | / |
|  | 680008528 | No | G | C | G | / | / | / |
| HORVU4Hr1G072130 | 583581644 | No | C | T | C | / | / | / |
|  | 583581673 | No | G | T | G | / | / | / |
|  | 583581707 | No | T | C | T | / | / | / |
|  | 583581733 | No | A | T | A | / | / | / |
|  | 583582358 | Yes | G | A | G | A | V | A |
| HORVU2Hr1G110130 | 718986261 | No | C | A | C | / | / | / |
|  | 718986460 | No | G | A | G | / | / | / |
|  | 718986613 | Yes | G | A | G | L | L | L |
|  | 718987999 | Yes | C | T | C | T | T | T |
| HORVU5Hr1G094280 | 594828539 | No | G | T | G | / | / | / |
|  | 594828556 | No | G | A | G | / | / | / |
|  | 594828585 | Yes | G | A | G | A | A | A |
|  | 594828747 | Yes | C | G | G | H | Q | H |
|  | 594828852 | Yes | C | T | C | I | I | I |
|  | 594829094 | Yes | C | T | C | T | I | T |
|  | 594829500 | Yes | T | C | T | A | A | A |
|  | 594829762 | Yes | T | C | T | * | Q | * |
|  | 594829832 | Yes | T | A | T | V | E | V |
|  | 594829852 | Yes | G | A | G | A | T | A |
|  | 594829902 | Yes | A | G | A | / | / | / |
|  | 594830013 | Yes | C | A | C | / | / | / |
|  | 594830045 | Yes | CAGT | C | CAGT | / | / | / |
|  | 594830061 | Yes | A | G | A | / | / | / |
| HORVU1Hr1G094880 | 556692068 | No | G | C | G | / | / | / |
|  | 556692087 | No | C | T | C | / | / | / |
|  | 556692426 | No | A | G | A | / | / | / |
| HORVU7Hr1G116630 | 643840898 | No | T | C | T | / | / | / |
|  | 643840925 | No | A | G | A | / | / | / |
|  | 643841021 | No | A | G | A | / | / | / |
|  | 643841058 | No | T | G | T | / | / | / |
|  | 643841097 | No | A | G | A | / | / | / |
|  | 643845043 | No | C | T | C | / | / | / |
|  | 643841218 | No | C | T | C | / | / | / |
|  | 643841231 | No | C | T | C | / | / | / |
